# RFX transcription factors are essential for hearing in mice

**DOI:** 10.1038/ncomms9549

**Published:** 2015-10-15

**Authors:** Ran Elkon, Beatrice Milon, Laura Morrison, Manan Shah, Sarath Vijayakumar, Manoj Racherla, Carmen C. Leitch, Lorna Silipino, Shadan Hadi, Michèle Weiss-Gayet, Emmanuèle Barras, Christoph D. Schmid, Aouatef Ait-Lounis, Ashley Barnes, Yang Song, David J. Eisenman, Efrat Eliyahu, Gregory I. Frolenkov, Scott E. Strome, Bénédicte Durand, Norann A. Zaghloul, Sherri M. Jones, Walter Reith, Ronna Hertzano

**Affiliations:** 1 Department of Human Molecular Genetics and Biochemistry, Sackler School of Medicine, Tel Aviv University, Tel Aviv 69978, Israel; 2 Department of Otorhinolaryngology, School of Medicine, University of Maryland Baltimore, 16 South Eutaw Street Suite 500, Baltimore, Maryland 21201, USA; 3 Department of Special Education and Communication Disorders, University of Nebraska Lincoln, Lincoln, Nebraska 68583-0738, USA; 4 Department of Medicine, Division of Endocrinology, Diabetes and Nutrition, School of Medicine, University of Maryland Baltimore, Baltimore, Maryland 21201, USA; 5 Department of Physiology, College of Medicine, University of Kentucky, Lexington, Kentucky 40536-0298, USA; 6 Centre de Génétique et de Physiologie Moléculaire et Cellulaire, CNRS UMR 5534, Université Claude Bernard Lyon-1, 69622 Villeurbanne, France; 7 Department of Pathology and Immunology, University of Geneva Medical School, CH-1211 Geneva, Switzerland; 8 Department of Medical Parasitology and Infection Biology, Swiss Tropical and Public Health Institute, and University of Basel, 4051 Basel, Switzerland; 9 Institute for Genome Sciences, University of Maryland School of Medicine, Baltimore, Maryland 21201, USA; 10 Department of Genetics and Genomic Sciences, Institute for Genomics and Multiscale Biology, Icahn School of Medicine at Mount Sinai, New York, New York 10029, USA; 11 Department of Anatomy and Neurobiology, University of Maryland School of Medicine, Baltimore, Maryland 21201, USA

## Abstract

Sensorineural hearing loss is a common and currently irreversible disorder, because mammalian hair cells (HCs) do not regenerate and current stem cell and gene delivery protocols result only in immature HC-like cells. Importantly, although the transcriptional regulators of embryonic HC development have been described, little is known about the postnatal regulators of maturating HCs. Here we apply a cell type-specific functional genomic analysis to the transcriptomes of auditory and vestibular sensory epithelia from early postnatal mice. We identify RFX transcription factors as essential and evolutionarily conserved regulators of the HC-specific transcriptomes, and detect *Rfx1,2,3,5* and *7* in the developing HCs. To understand the role of RFX in hearing, we generate *Rfx1/3* conditional knockout mice. We show that these mice are deaf secondary to rapid loss of initially well-formed outer HCs. These data identify an essential role for RFX in hearing and survival of the terminally differentiating outer HCs.

## Abstract (web summary)

Inner ear hair cells are non-regenerative mechanosensory cells essential for hearing. Here, with cell-type-specific expression analyses, the authors identify RFX transcription factors as central mediators of their survival during terminal differentiation and thus essential for hearing in mice.

## Graphical abstract

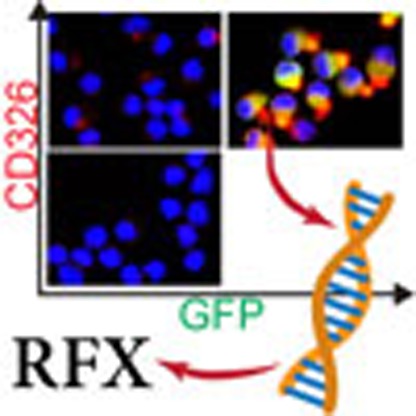

Sensorineural hearing loss affects 1:500 newborns^1^ and the majority of the elderly population^2^. The sensations of sound and movement are dependent on highly specialized post-mitotic mechanosensory cells called hair cells (HCs)^3^. Mammalian auditory HCs do not regenerate and their loss is a final common pathway in most forms of hearing dysfunction^4^. For this reason, understanding the molecular signalling cascades that lead to HC differentiation is important for hearing restoration. To date, several master regulators of HC fate and differentiation have been characterized. Among these are the transcription factors (TFs) ATOH1 (ref. 5), POU4F3 and GFI1 (refs 6, 7, 8). Nevertheless, forced expression of these three TFs in stem cells leads only to immature hair-cell-like cells^9^, underscoring the need to identify factors that mediate the differentiation and survival of maturating HCs. Furthermore, while the auditory and vestibular HCs and supporting cells (SCs) are structurally and functionally distinct, very few molecular differences between these cell types have been reported. Detailed knowledge of such markers, as well as regulators of terminal differentiation, is important to identify genes with a role in hearing and balance.

Gene expression analysis has been applied successfully to study development^10^,^11^, regeneration^12^,^13^, and identification of transcriptional cascades and molecular signalling pathways in the ear^14^. Given the complex structure of the inner ear sensory epithelia, cell type-specific analyses, either in the form of population analysis of sorted cells or in the form of single-cell analysis, have grown in favour^14^,^15^,^16^.

Here by performing a comprehensive cell type-specific comparison of the transcriptomes of HCs to other cell types from the auditory and vestibular systems of early postnatal mice, we identify the Regulatory Factor X (RFX) family of transcription factors as a key regulator of HC transcriptomes. Our results indicate an evolutionarily conserved role for RFX TFs in regulating the expression of genes encoding HC-enriched transcripts and demonstrate that RFXs are necessary for hearing in mice. In addition, we show that contrary to the known role of RFX as major regulators of cilia formation^17^, in RFX1/3 deficient HCs, the primary cilia (kinocilia) develop, and planar cell polarity is not impaired. The newly formed HCs seem structurally normal and functional until the outer HCs (OHCs) die rapidly at the onset of hearing, the time when the kinocilia are normally retracted. These data support a novel role for RFX in hearing, by maintaining the survival of normally formed HCs, probably through the regulation of their transcriptome during terminal differentiation.

## Results

### Inner ear cell type-specific gene clusters

**Figure 1:**
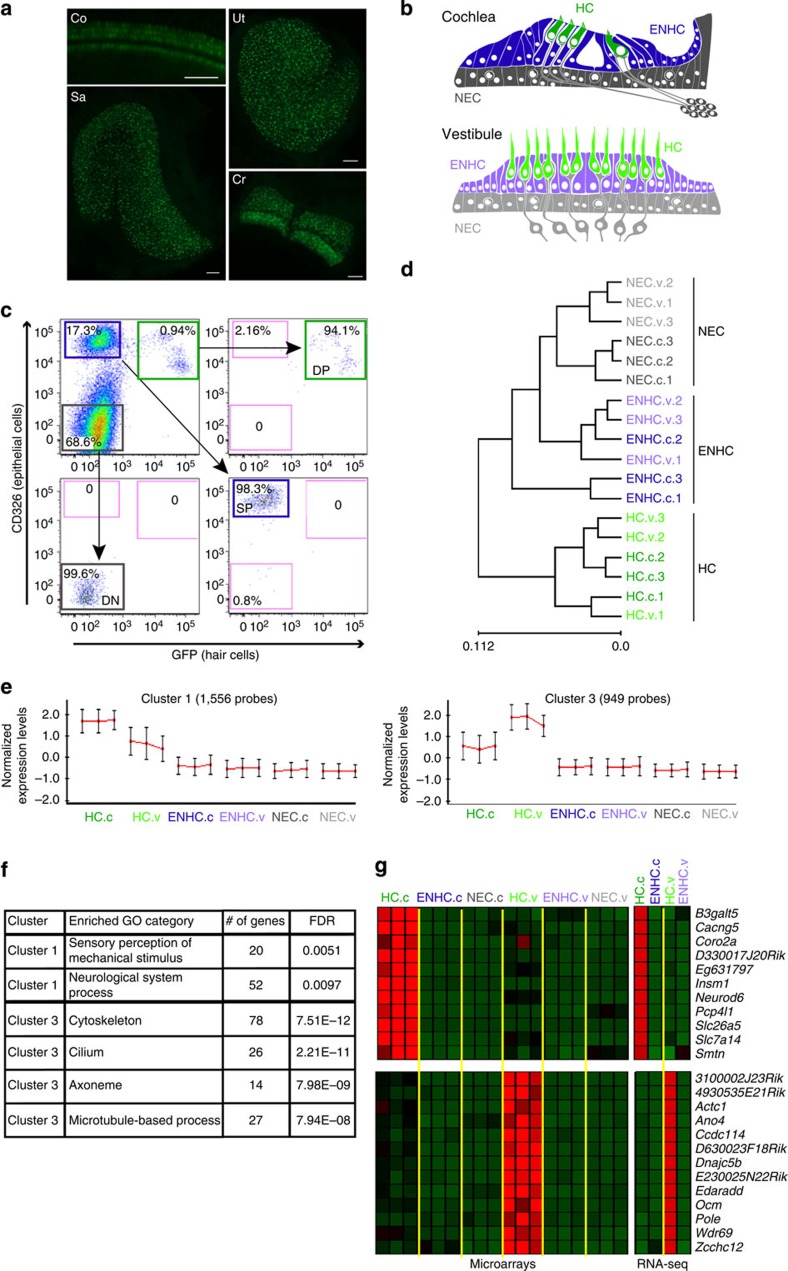
HC transcriptome analysis. (**a**) Representative images from an inner ear of an *Atoh1/nGFP* mouse. All HCs in the auditory and vestibular systems are GFP(+). Co-cochlea; Cr-crista; S-saccule; U-utricle. Scale bar, 50 μm. (**b**) Schematic diagram of cochlear and vestibular sensory epithelia, illustrating the cells designated as HC, ENHC and NECs in each organ. (**c**) Representative image of flow cytometry from cochlear epithelia of newborn *Atoh1/nGFP* mice. Left upper quadrant—pre-sorting figure: HCs are double positive (DP), ENHCs are positive for CD326 (SP), NECs are negative for CD326 and GFP (DN). Post-sort analyses of sorted cells showing a cell type-specific purity >94% for HCs, >99% for NECs and >98% for ENHCs. (**d**) Hierarchical clustering, based on all the expressed genes in the data set, separated the probed samples into three major branches according to cell type: HC, NEC and ENHC (c-cochlea and v-vestibular samples). (**e**) Two major clusters containing genes whose expression is elevated in HCs. Each cluster is represented by the mean pattern of its genes’ expression levels (error bars: ±s.d. calculated over all genes assigned to each cluster). Expression levels of each gene were standardized (mean=0, s.d.=1) prior to clustering. This methodology enabled grouping of genes that share similar patterns, but not necessarily the same magnitude of expression. The entire set of clusters is shown in Supplementary Fig. 2. (**f**) Enriched GO categories with a *P* value <0.01 (hypergeometric test) after false discovery rate correction for multiple testing. (**g**) Marker genes specific for cochlear HCs (top) and vestibular HCs (bottom) as detected in the expression microarray data set (left) and confirmed by the RNA-seq data set (right). Red represents enrichment, Green represents depletion. Data represent results from three biological replicates.

To characterize the HC transcriptome in early post-natal auditory and vestibular systems, we used the *Atoh1/nGFP* transgenic mice expressing a green fluorescent protein (GFP) in all inner ear HCs^18^ (Fig. 1a,b). Auditory and vestibular epithelia from inner ears of postnatal day 1 (P1) mice were separated into HCs, epithelial non-HCs (ENHCs) and non-epithelial cells (NECs) by flow cytometry (Fig. 1c, Supplementary Fig. 1). Gene expression levels were recorded from the sorted cells using whole genome expression microarrays (Supplementary Data 1). Hierarchical clustering, applied to all genes detected as expressed, showed a clear division of the samples based on cell types, namely HC, ENHC and NEC (Fig. 1d), demonstrating, as expected, that cell-type identity, rather than tissue of origin, is the major determinant of the cell transcriptome.

To define patterns of gene expression, we first searched for differentially expressed genes using an analysis of variance. We identified 6,556 probes, representing 4,269 unique genes (false discovery rate<5%) as differentially expressed between the cell types and tissues. Cluster analysis applied to this set of differentially expressed genes detected 12 main expression patterns (Supplementary Fig. 2). The genes with a higher level of expression in HCs were divided into a cochlear-enriched cluster (cluster 1) and a vestibular-enriched cluster (cluster 3) (Fig. 1e). Functional enrichment analysis revealed that the cochlear HC cluster is significantly enriched for genes that regulate sensory perception of mechanical stimuli, whereas the vestibular HC cluster is significantly enriched for cytoskeleton and cilium-related genes (Fig. 1f).

In parallel, we used RNA-seq to record expression profiles of cochlear and vestibular HCs and ENHCs (Supplementary Data 2). There was a strong correlation between expression patterns measured by microarrays and RNA-seq, with RNA-seq measurements showing an improved dynamic range (Supplementary Fig. 3a,b). Among the genes detected by RNA-seq, the expression of 2,176 was elevated at least threefold in HCs compared with ENHCs in either the auditory or vestibular systems. In accordance with the patterns detected by microarray, the RNA-seq data set revealed two major expression clusters containing genes with increased expression in both auditory and vestibular HCs (clusters 1 and 2, Supplementary Fig. 4a,b). Moreover, the RNA-seq analysis detected not only HC-enriched protein-coding transcripts but also a multitude of long, non-coding transcripts whose expression is elevated in HCs (Supplementary Data 2).

To determine whether differential expression can be predictive of functional significance, we examined the expression of mouse orthologues of the known human deafness-causing genes detected in the microarray and RNA-seq data sets. Interestingly, out of 70 orthologues of known human deafness-causing genes, 56 and 64 were detected as expressed in at least one cell type, in the microarray and RNA-seq, respectively. Of these, in the microarray data set, 41 genes were differentially expressed (enrichment *P* value=1.6E−9; hypergeometric test), and statistically over-represented in the clusters of genes whose expression is elevated in HCs (29 genes, *P* value=5.5E−6; hypergeometric test). Similarly, in the RNA-seq, 33 genes showed at least threefold elevated expression in HCs compared with ENHCs in either the auditory or vestibular systems (*P* value=4.2E−11; hypergeometric test; Supplementary Data 3; and see examples in Supplementary Fig. 4c). With ∼100 deafness loci remaining to be cloned, these data suggest that differential inner ear expression and specifically elevated expression in HCs, could be used as a valuable criterion in prioritizing genes/variants for validation.

### Identification of inner ear cell type-specific markers

Next, to further define cell type-specific marker genes within each tissue (namely genes whose expression is strictly confined to a particular cell-type in our examined panel), we applied stringent expression criteria to the microarray data set (Methods). This analysis identified 158 annotated genes as markers of inner-ear HCs (out of 276 probes corresponding to annotated and predicted genes; Supplementary Data 4). Most of these genes marked both the cochlear and vestibular HCs, and included many genes that, in the newborn mouse inner ear, are known markers of HCs, such as *Atoh1*, *Pou4f3* and *Gfi1*. *Egfp* was identified as a HC-specific marker validating the specificity of the sorting approach. Of interest, 11 genes were specific for the cochlear HCs while 13 were specific for the vestibular HCs (Fig. 1g). These included *Slc26a5*, a known cochlear HC-specific marker (uniquely expressed in OHCs^19^), and *Ocm* which in the newborn inner ear is not detected in cochlear HCs but is already expressed in vestibular HCs^20^. Similarly, we identified 32 unique genes as markers of inner ear ENHCs of which 7 were specific for the cochlea and 4 were specific for the vestibular system (Supplementary Data 4).

To validate the identified cell type-specific markers, we examined our RNA-seq measurements and found a remarkable confirmation for the specificity of our marker sets (Fig. 1g; Supplementary Fig. 5). Specifically, of the 158 genes defined by the microarray as markers of HCs, 155 showed in the RNA-seq data at least 2-fold enrichment in HCs, with an average of 10-fold enrichment in cochlear HCs and 14-fold enrichment in vestibular HCs (Supplementary Data 4). As for the set of ENHC markers, for all except 1 gene, the expression levels measured by RNA-seq were at least 2-fold enriched in ENHCs compared with HCs (with an average of 11.3-fold and 7.5-fold for the cochlear and vestibular ENHCs, respectively). The stringent criterion we set for calling genes as HC-markers favours specificity (low rate of false positives) and over sensitivity (low rate of false negatives), and accordingly many genes whose expression is enriched in HCs were not called as marker genes (Supplementary Fig. 5). Overall, these data define over 150 validated HC and ENHC cell type-specific and tissue-specific markers in the newborn mouse inner ear.

### RFX are important regulators of the HC transcriptome

**Figure 2:**
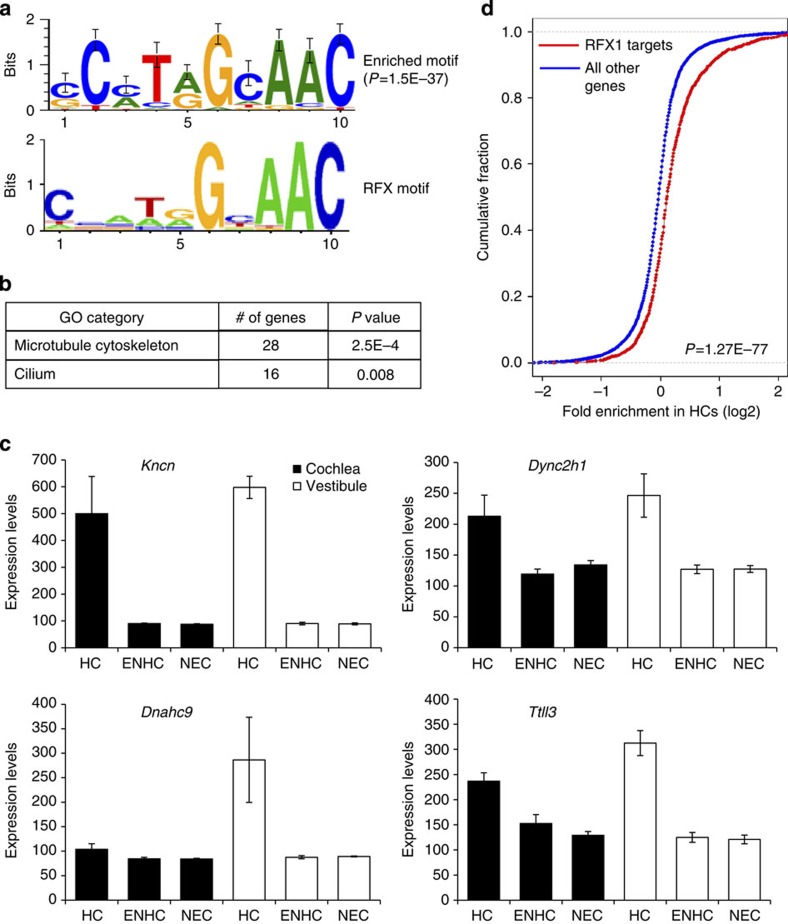
RFX binding signature is enriched in promoters of HC-enriched genes. (**a**) *De-novo* motif analysis revealed that the promoters of genes whose expression is elevated in inner ear HCs are significantly enriched for a DNA motif that matches the binding signature of the RFX family of TFs (the known RFX motif is from TRANSFAC DB^70^; accession number M00281). The enriched motif was detected in promoters of 225 HC-elevated genes (enrichment factor=2.7; *P* value=1.5E−37). (**b**) GO functional categories that were over-represented in the set of 225 putative RFX HC target genes. (**c**) Expression pattern of selected putative RFX targets that function in ciliogenesis (*n*=3; error bars: ±s.d.). (**d**) HC enrichment was defined per gene as the ratio between its expression in HCs and its average expression over the three probed cell types (HC, ENHC and NEC). The plot compares the cumulative distribution of these HC-enrichment measures (in log2 scale; *x*-axis) between the set of all RFX1 targets defined by ChIP-seq analysis (red) and all the other genes in the data set (blue). The distribution for the RFX1 targets is significantly shifted to the right, demonstrating that their expression level as a group is markedly elevated in HCs (*P* value=1.3E−77; Wilcoxon test). See Supplementary Fig. 9b for similar analysis applied to ChIP-Seq targets of RFX3.

Our next goal was to identify transcription factors that regulate the expression of genes encoding HC-enriched transcripts. We and others have demonstrated that computational analysis of *cis*-regulatory elements in promoters of differentially expressed genes is a powerful tool for the identification of transcriptional regulators^10^,^21^,^22^. This reverse-engineering approach for delineating transcriptional networks is based on the assumption that genes co-expressed in the same cell-type(s) are likely to be regulated by common transcription factors that are specifically active in those cells. Thus, these genes are expected to share specific DNA regulatory elements in their promoter regions. Therefore, we computationally searched for DNA motifs whose prevalence in the promoters of genes encoding HC-enriched transcripts, as detected by our microarray and RNA-seq data, is significantly higher than in the promoters of all other genes in our data set. We defined promoters as the genomic region from 1,000 nucleotides (nt) upstream to 500 nt downstream of the transcription start site (TSS). A single, highly statistically significant DNA motif was identified as over-represented in promoters of the genes encoding HC-enriched transcripts. This motif corresponds to the binding signature of the RFX family of transcription factors (Fig. 2a), and its over-representation was most prominent on promoters of genes encoding HC-enriched transcripts, which showed higher elevated expression in vestibular compared with auditory HCs (cluster 3 in Fig. 1e and cluster 1 in Supplementary Fig. 4a). The RFX signature was detected on promoters of 225 genes encoding HC-enriched transcripts, resulting in a ∼3-fold over-representation of the motif (*P* value=1.5E−37; hypergeometric test) and tagging the corresponding genes as putative direct targets of RFX in HCs (Supplementary Data 5). A similar sequence analysis using promoters of the human and zebrafish orthologous genes also pinpointed the RFX motif as highly enriched and the top scoring motif in both organisms, suggestive of an evolutionarily conserved pathway (enrichment *P* value=2.9E−30 and 9.2E−29 for human and zebrafish promoters, respectively; hypergeometric test; Supplementary Fig. 6). Notably, the putative targets include *Lrrc51*, a known deafness-causing gene^23^, *Ift88* (ref. 24)*, Wdpcp*^25^*, Tmie*^26^,^27^*, Ush2a* and *Ush1c*^28^, all of which have known roles in murine HC development. Consistent with the known role of RFX proteins as master regulators of ciliogenesis^29^, the 225 putative RFX target genes were enriched for cytoskeleton and cilium genes (Fig. 2b,c). Overall, the expression of the putative RFX targets was significantly enriched in both the cochlear and the vestibular HCs, with the vestibular showing a greater level of elevated expression (Supplementary Fig. 7).

We next analysed two recent expression data sets that also recorded gene expression in the inner-ear and defined sets of HC-enriched genes. One of these studies purified cochlear HCs using the *Atoh1*^*A1GFP/A1GFP*^ mouse^30^, and the other used *Pou4f3-GFP* mice to isolate auditory and vestibular HCs for expression analysis^31^. Notably, in both of these data sets we detect the RFX binding signature as the most highly over-represented motif in the promoters of genes encoding for HC-enriched transcripts (Supplementary Fig. 8a). We also find that the expression of the set of putative RFX target genes derived from our combined microarray and RNA-seq data sets is highly enriched in HCs as measured by these two independent studies (Supplementary Fig. 8b,c).

### RFX targets identified by ChIP-seq are elevated in HCs

To validate and further identify which of the candidate target genes are bound *in vivo* by RFX, we analysed a chromatin-immunoprecipitation sequencing (ChIP-seq) data set performed with antibodies for RFX1 and RFX3 in the mouse Min6 cell line^32^. In this data set, RFX1 and RFX3 were found to bind the promoters of 2,540 and 3,575 genes, respectively, with a highly significant overlap (Supplementary Fig. 9a). We found that as a group, the expression of ChIP-seq-identified RFX target genes was significantly elevated in HCs compared with their expression in ENHCs and NECs (Fig. 2d; Supplementary Fig. 9b). This result further supports the computational prediction of a role for RFX in regulation of gene expression in HCs. Moreover, intersection between the computationally predicted RFX targets in HCs and the ChIP-seq data confirmed 142 of the 225 candidate promoters as bound *in vivo* by at least 1 of the probed RFX TFs and 130 of these promoters were bound by both RFX1 and RFX3, indicating a high degree of overlap in DNA binding between RFX members in the HCs (Supplementary Data 5).

### The role of RFX in HCs is evolutionarily conserved

**Figure 3:**
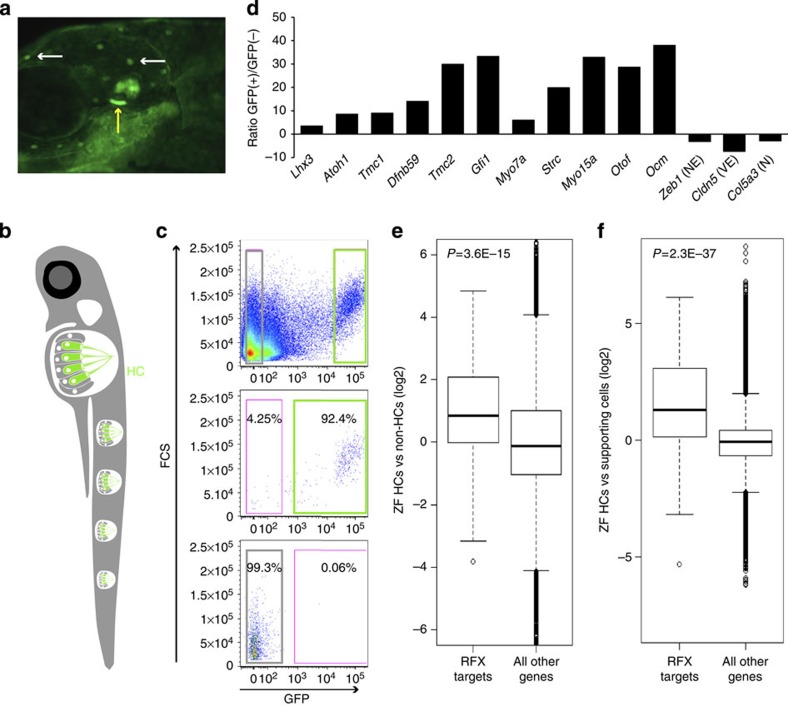
The expression of RFX target genes is elevated in zebrafish HCs. (**a**) An image of a 5 dpf *ppv3b:GFP* larvae showing GFP expression in the inner ear HCs (yellow arrow) and in the neuromast HCs (white arrows). (**b**) Schematic representation of a zebrafish showing the neuromast HCs. (**c**) Representative image of flow cytometry from dissociated 5 dpf *ppv3b*:GFP larvae. Top—dot plot analysis reveals a distinct population of GFP(+) cells (right). Post-sort analyses showing a cell type-specific purity >92% for GFP(+) cells (middle panel), >99% for negative cells (bottom). (**d**) The expression of zebrafish orthologues of known murine HC-enriched genes. Expression is enriched in the zebrafish GFP(+) cell population, while markers of NECs (for example, *Zeb1*), vascular epithelial cells (VE; for example, *Cldn5*) and neuronal cells (N; for example, *Col5a3*) are decreased (results based on RNA-seq). (**e**) Expression of the zebrafish orthologous genes of the 225 putative targets of RFX that we identified in murine HCs, was significantly elevated in zebrafish HC (GFP(+) cells). Ratios between expression level in the GFP(+) and GFP(−) cells were calculated for each gene in the zebrafish data set. The plot compares the distribution of these ratios (in log2 scale; *y*-axis) between the set of orthologues of the RFX targets (left boxplot) and all the other genes in the data set (right boxplot). The box indicates the first and third quartiles; the horizontal band inside the box indicates the median. The whiskers extend to the most extreme data point, which is no more than 1.5 times the interquartile range from the box. *P* value calculated using Wilcoxon test. (**f**) Similar to panel (**e**), but using an independent zebrafish gene expression data set. Here too, expression of RFX target genes is significantly elevated in HCs as compared with their expression levels in non-HCs.

We extended our analysis to another species, to determine whether the role of RFX in regulating the HC transcriptome is evolutionarily conserved, and utilized the *ppv3b:GFP* transgenic zebrafish, which express GFP predominantly in HCs^33^ (Fig. 3a,b). We sorted GFP-positive (+) and negative (−) cells from 5-day post fertilization (dpf) larvae using flow cytometry (Fig. 3c) and profiled their transcriptomes using RNA-seq (Supplementary Data 6). We observed a 3- to 37-fold enrichment for known HC-enriched transcripts in the GFP(+) cells, confirming their identity as HCs (Fig. 3d). Consistent with an evolutionarily conserved role for RFX in regulating the HC transcriptome, expression of the zebrafish orthologues of the 225 putative RFX target genes identified in mouse was significantly elevated in the zebrafish GFP(+) cells (Fig. 3e). Conversely, zebrafish orthologues of mouse inner ear vascular endothelial- or neuronal-enriched transcripts were not elevated in the zebrafish GFP(+) cells, validating the specificity of our result (Supplementary Fig. 10a,c).

As a means of independent corroboration, we analysed a published data set of zebrafish HCs compared with SC gene expression, generated from 5 dpf *alpl:mCherry;pou4f3:GFP* larvae^34^. Again, the RFX motif was the only statistically over-represented signature detected in the promoters of genes with enriched expression in zebrafish HCs (Supplementary Fig. 10d). In this data set too, expression of the zebrafish orthologues of our 225 putative RFX targets was significantly elevated in HCs (Fig. 3f).

### RFX transcription factors are essential for hearing

**Figure 4:**
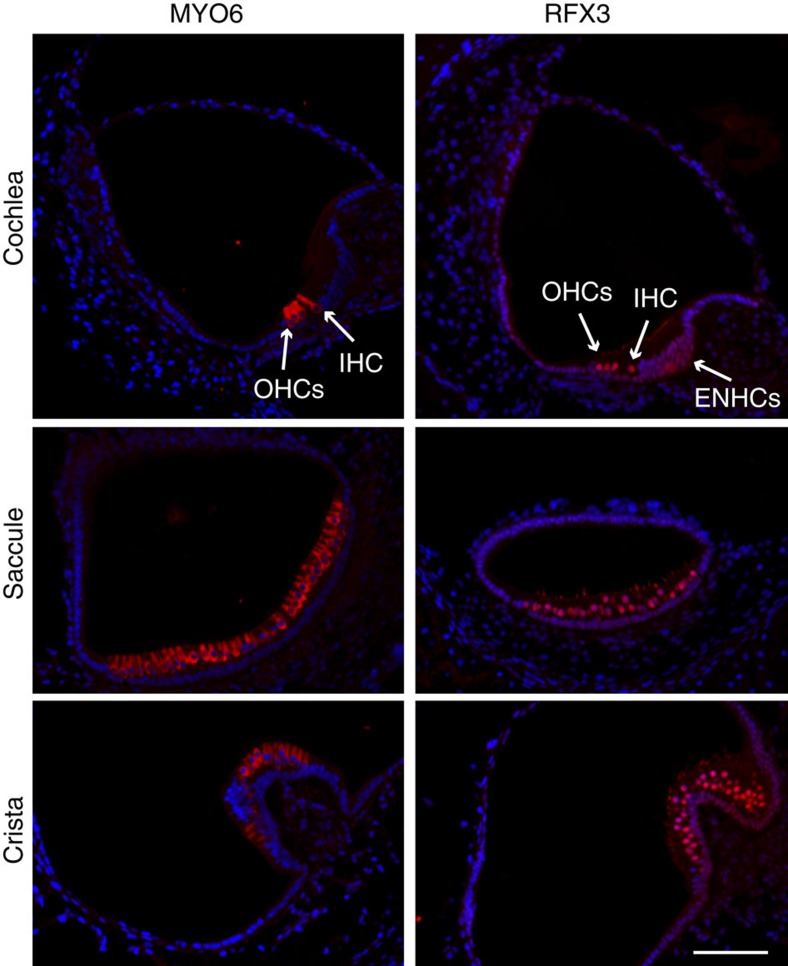
RFX3 expression in the mouse inner ear. Inner ear sections from a P1 wild-type mouse stained with an antibody for MYO6, which marks the inner ear hair cells (left panel) and RFX3 (right panel), in red, and counterstained with DAPI, in blue. A robust nuclear expression of RFX3 is detected in all HCs with a much weaker expression in ENHCs. Staining of the stereocilia with the RFX3 antibody is non-specific, validated by staining cKO ears in which the nuclear staining is abolished and the stereocilia staining persists (Supplementary Fig. 11). IHC, inner hair cell; OHCs, outer hair cells. Representative images of *n*>5 experiments. Scale bar, 60 μm.

**Figure 5:**
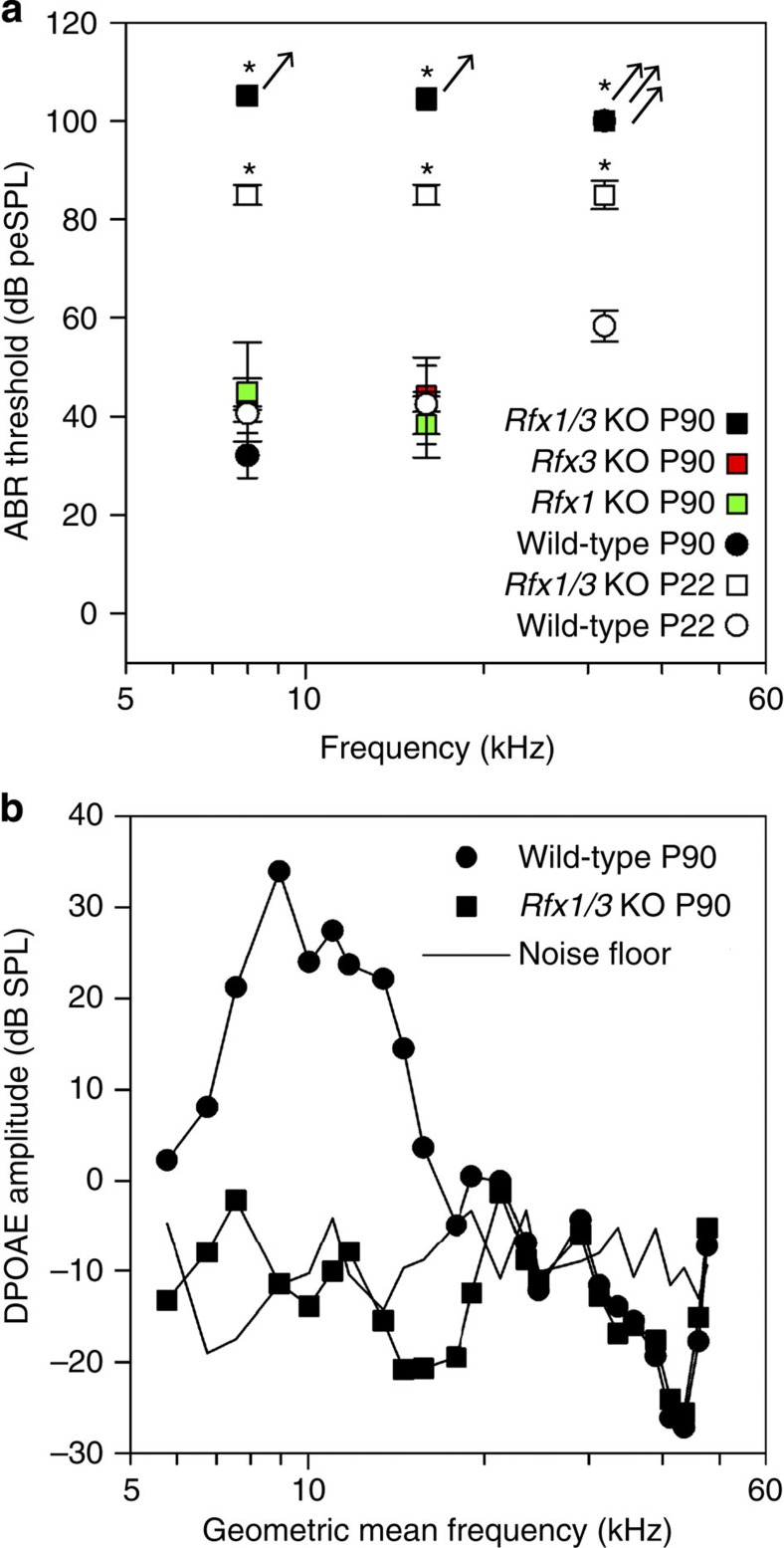
*Rfx* genes are necessary for hearing. (**a**) Elevated hearing thresholds in the *Rfx1/3* cKO mice: 3-month-old *Rfx1* cKO and *Rfx3* cKO have hearing thresholds indistinguishable from their wild-type littermate controls at 8 and 16 kHz. However, *Rfx1/3* cKO mice have significantly elevated hearing thresholds compared with their age-matched controls already at postnatal day 22. Their hearing thresholds progress to a profound hearing loss as measured at P90. All strains exhibited elevated thresholds at 32 kHz at P90. Arrows indicate that mean thresholds are likely higher than those indicated because a number of animals had absent ABR at the maximum stimulus levels tested. Error bars are ±s.e.m. ‘*’ are *P* value<0.01 (multivariate ANOVA test for P22 data and ANOVA test for P90 data). (**b**) Distortion Product Otoacoustic Emissions (DPOAE) amplitudes, indicative of outer HC function, were also significantly reduced for *Rfx1/3* cKO mice compared with wild-type controls. The following genotypes were tested at P22: *Rfx1/3* cKO (*n*=4), wild type (*n*=10); and at P90: *Rfx1* cKO (*n*=3), *Rfx3* cKO (*n*=8), *Rfx1/3* cKO (*n*=10), wild type (*n*=6).

To determine whether RFX are necessary for hearing, we first queried our RNA-seq data and found that *Rfx3* is enriched in HCs and the most abundant RFX transcript in the inner ear (Supplementary Data 2). In addition, immunostaining detected a robust nuclear expression of RFX3 in all HCs of the auditory and vestibular systems along with much weaker expression in ENHCs (Fig. 4, Supplementary Fig. 11). Therefore, RFX3 is likely to be an important TF-regulating HC development. To determine the role of RFX proteins in HCs, we generated conditional knockout (cKO) mice. First, we used the *Gfi1-Cre* mice to delete *Rfx3* from all inner ear HCs. Hearing measurements of 3-month-old *Rfx3* cKO revealed normal thresholds which could, however, represent functional redundancy with other RFX family members (Fig. 5). We next examined which additional RFX family members are expressed in HCs and detected expression of *Rfx1*, *Rfx2*, *Rfx5* and *Rfx7*. We generated cKO for *Rfx1* (Supplementary Fig. 12), because RFX1 and RFX3 share conserved structural domains^35^, show a high degree of overlap in their DNA binding to candidate genes, and form heterodimers in many cell types^36^,^37^. As with *Rfx3* cKO, we observed normal hearing in the *Rfx1* cKO (Fig. 5). However, remarkably, the *Rfx1/3* double cKO mice suffered from rapidly progressive hearing loss with significantly elevated hearing thresholds already at P22 progressing to no measurable hearing by 3 months of age (Fig. 5).

### Rapid OHC loss in *Rfx1/3* cKO mice

**Figure 6:**
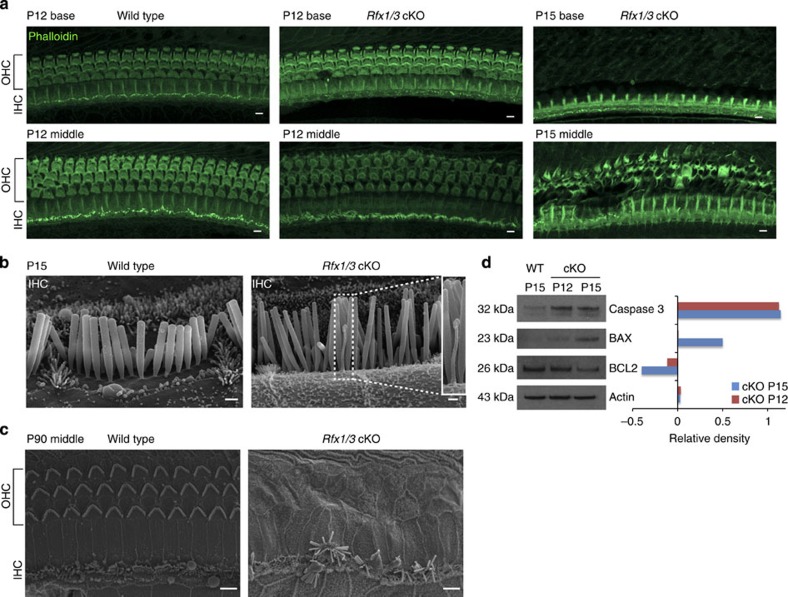
Rapid degeneration of the terminally differentiating OHCs in the *Rfx1/3* cKO mice. (**a**) Confocal imaging of the basal and middle turns of cochlear ducts from wild-type (P12) and *Rfx1/3* cKO (P12 and 15) stained with phalloidin (green), *n*=4 for all genotypes. A diffuse loss of OHCs is seen in the P15 cKO mice progressing from the basal to the middle turn. (**b**) Lateral view of IHC of P15 mice, demonstrating the retained kinocilium in the IHC of the mutant mice. Inset shows a high magnification image of the retained kinocilium. (**c**) SEM of a middle turn from 3-month-old mice showing complete degeneration of the OHCs and disrupted IHCs in the double cKO mice. *N*=4 for all genotypes. Scale bars for **a**,**c**: 5 μm; for **b**: 0.5 μm. (**d**) Western blot analysis was performed to detect changes in expression of markers for cell death and survival in cochlear ducts dissected from P15 wild-type (controls) and P12 and P15 *Rfx1/3* cKO mice (left panel). Densitometry analysis was performed to quantify the changes in protein expression relative to the levels in the wild-type tissue (right panel). Results show a significant increase in caspase 3 and BAX along with a decrease in BCL2 consistent with cell death. Actin was used as a loading control. Left margin indicates the molecular weight. Each lane consists of tissue obtained from four ears. This set of experiments was performed twice using biologically independent samples and a representative figure is shown.

To determine the cause of the hearing loss in the *Rfx1/3* double cKO mice, we performed whole mount phalloidin staining and Scanning Electron Microscopy (SEM). In the mutant mice, the sensory epithelium appeared normal at P12 throughout the length of the cochlear duct, with no significant loss of HCs (Fig. 6a). In contrast to P12, there was a widespread loss of outer but not inner HCs by P15, consistent with the elevated hearing thresholds at P22 (Fig. 5a). In addition, in contrast to wild-type inner HCs (IHCs), *Rfx1/3* cKO IHCs had retained kinocilia at P15 (Fig. 6b), suggesting that RFX1/3 are involved in normal retraction of this organelle in the late development, at least in IHCs. By P90, we observed near complete loss of all OHCs throughout the length of the cochlear duct and progressive IHC degeneration associated with sporadic fusion of stereocilia on some of the remaining IHC stereociliary bundles (Fig. 6c).

To further clarify the mechanism of the loss of OHCs, we analysed the expression of select cell death markers by western blot analysis. We detected a decrease in the expression of BCL2 (pro-survival factor), along with an increase in caspase-3 and BAX (pro-apoptotic factors), in the P12 and P15 mutant cochlea compared with P15 controls (Fig. 6d, Supplementary Fig. 13). Thus, despite the intact appearance of the sensory epithelium, cell death has already initiated at P12, leading to a rapid and diffuse loss of OHCs by P15. These data indicate that a lack of RFX expression leads to programmed cell death in the OHCs.

### Early differentiation of OHCs in *Rfx1/3* cKO mice is normal

**Figure 7:**
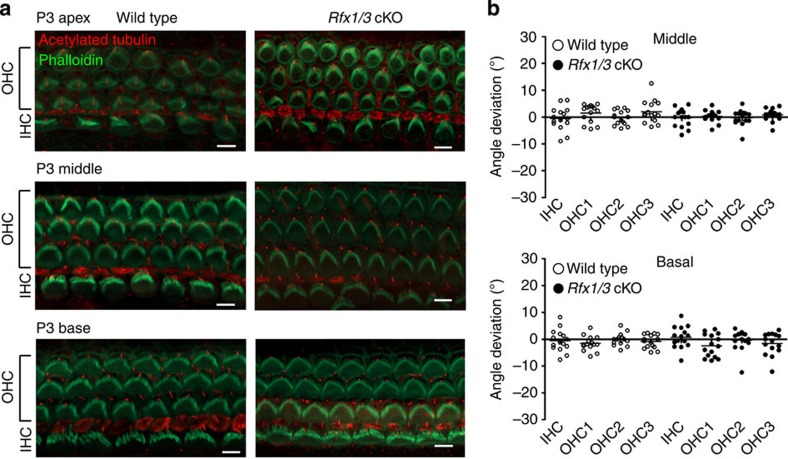
The stereociliary bundles of the HCs in the *Rfx1/3*cKO mice are properly polarized. (**a**) Representative confocal microscopy images from the basal, middle and apical turns of 3-day-old *Rfx1/3* cKO mice (*n*=3) and their wild-type littermate controls (*n*=3). Cochleae were stained with phalloidin (green) to define the actin cytoskeleton and stereociliary bundles, and an antibody for acetylated tubulin (red) to define the kinocilium. The stereociliary bundles of the wild-type and mutant mice appear properly polarized in the basal and middle turns. In the apical turn at P3, the polarity of the bundles is less organized in both the wild-type and mutant mice. Scale bar, 5 μm. (**b**) Quantification of the angle of deviation of the bundles in the wild-type and mutant mice, as measured from a line perpendicular to the axis of the pillar cells, crossing through the middle of the HCs. Results from the basal and middle turns are represented in a scatter dot plot. Each dot represents a HC, horizontal bars represent the median value. The polarity of five consecutive IHCs, first row OHCs, second row OHCs and third row OHCs was measured from cochleae of three separate mice from each genotype and from two regions: basal and middle turns, separately. A one-way ANOVA followed by a Tukey’s test was used to compare the results. No statistical significant differences were detected (*P* value>0.05).

**Figure 8:**
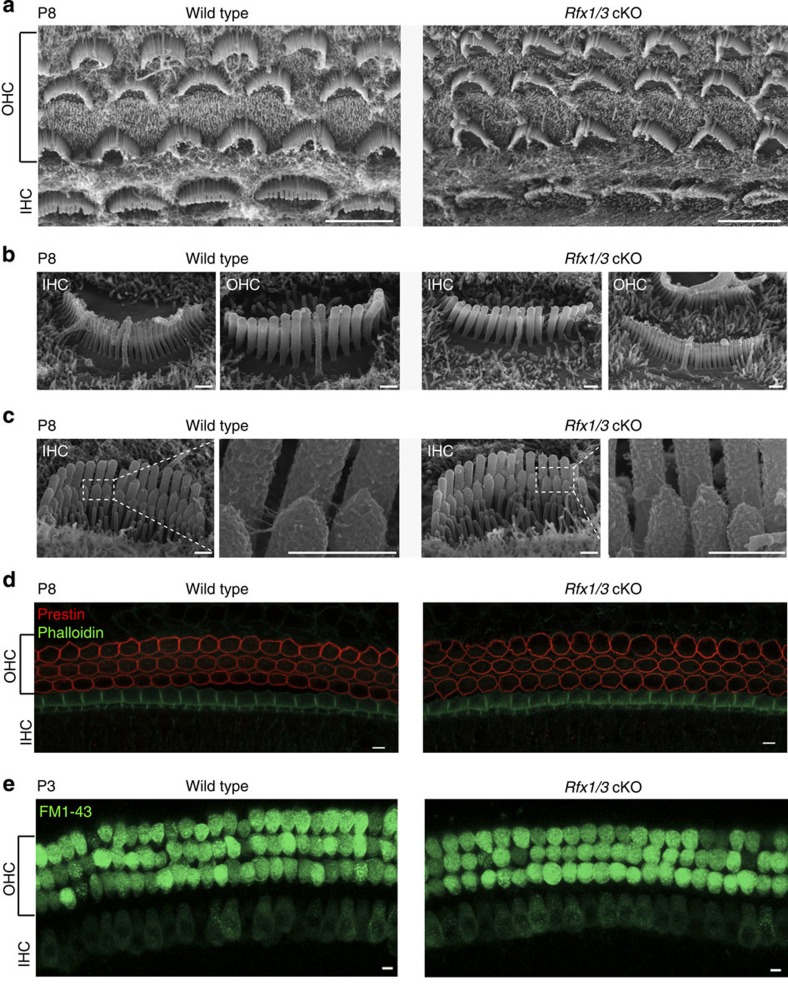
*Rfx1/3* are not necessary for the early differentiation of OHCs. (**a**) SEM images of the middle turns from P8 *Rfx1/3* cKO mice and their wild-type controls showing normal appearance of the cochlear sensory epithelia with one row of IHCs and three rows of OHCs. All HCs have kinocilia. (**b**) Higher magnification lateral views of IHCs and OHCs of *Rfx1/3* cKO and littermate P8 controls showing properly formed kinocilia. (**c**) Higher magnification medial views of IHCs; Inset show the tip links at higher magnification from the regions indicated in the dashed boxes. (**d**) Basal turns of P8 cochlear ducts from *Rfx1/3* cKO and controls stained with an antibody for prestin (red) and phalloidin (green). All OHCs express prestin in their lateral wall. (**e**) A functional assay for the presence of an intact transduction channel was performed using the uptake of FM1-43 dye. HCs throughout the cochleae of the wild-type and double cKO mice internalized the dye within 10 s. Scale bars for **a**,**d**,**e**: 5 μm; Scale bars for **b**,**c**: 0.5 μm. *n*=3 for P8 SEM analysis, *n*=4 for prestin staining and *n*=8 for FM1-43 analysis.

As we observed an accelerated degeneration of HCs at P15, we next performed structural and functional studies to examine the HCs in the earlier postnatal period. We first evaluated the HC polarity using confocal microscopy, because the RFX TFs are associated with ciliogenesis^17^. Abnormalities of ciliogenesis in the inner ear usually result in the disruption of planar cell polarity^38^. We found that *Rfx1/3* cKO stereocilia bundles were properly polarized throughout the length of the cochlear duct (Fig. 7). We further evaluated the structure of the hair bundles using SEM and found that *Rfx1/3* cKO HCs at P8 have well-formed bundles, including intact primary cilia (kinocilia), stereocilia, tip-links and inter-stereociliary links (Fig. 8a,c). To determine whether OHCs express markers associated with functional maturation in the first postnatal week, we stained cochleae for the OHC-specific molecular motor prestin^19^, and found an identical expression of prestin in the mutant and wild-type mice (Fig. 8d). Finally, consistent with functional mechanotransduction^39^, the newborn HCs of the *Rfx1/3* cKO exhibited intact uptake of FM1-43 (Fig. 8e). These data demonstrate that early development and maturation of HCs is not affected in the *Rfx1/3* cKO, and identify a critical role for RFX1/3 proteins in regulating the survival of the post-natal terminally differentiating OHCs.

## Discussion

In this study, we employed a genomic cell type-specific approach to comprehensively profile the HC transcriptome. Our data revealed numerous HC and ENHC marker genes, common and divergent between the auditory and vestibular systems at P1. We identified and validated 183 HC and ENHC cell type-specific markers, many of which have not been previously described in the ear. Several of these markers include genes encoding regulatory proteins (for example, *Insm1*, *Pou2af1*), ion channels (for example, *Ano4*), kinases (for example, *Fn3k*, *Nek5*) or ubiquitin ligases (for example, *Trim36*). The list also includes numerous genes with a known function in inner ear development. In addition, using an analysis of expression levels of genes encoding for homologues of known deafness-causing genes, we found that differential cell type-specific expression in the ear can be suggestive of functional significance. Taken together, and given the high stringent criteria used to identify these newborn inner ear cell type-specific markers, we anticipate that many of their encoding genes carry critical roles in inner ear development.

Gene expression levels are often not sufficient to identify key regulatory pathways, because numerous transcription factors are expressed in any given cell type, and the level of expression does not necessarily correlate with a functional significance. Using an unbiased reverse-engineering approach for *de novo* identification of transcriptional regulators of gene expression, we identified the family of RFX proteins as evolutionary conserved regulators of the HC transcriptome. Furthermore, analysis of the single cell RNA-seq data in the accompanying study by Burns *et al*.^40^ identifies RFX binding motifs as the most enriched in the set of genes whose expression increases in differentiating HCs, consistent with our results. We also detected the RFX-binding motif as the top scoring over-represented signature in promoters of sets of HC-enriched genes defined by two recently published studies. Taken together, these results point to a central role for RFX TFs in controlling the HC transcriptome.

Based on the role of RFX in ciliogenesis, we anticipated either the loss of kinocilia in the mutant HCs or functional defects associated with ciliogenesis—such as planar cell polarity defects^38^. The normal appearance of the HCs in the young postnatal *Rfx1/3* cKO mice was therefore surprising. In addition, the expression of the 225 predicted RFX target genes in the vestibular HCs at P1 is higher than the expression of these genes in the auditory HCs (Supplementary Fig. 7). Therefore, the normal behavioural vestibular phenotype in *Rfx1/3* cKO mice was also surprising. However, we cannot exclude possible compensatory function of other RFX genes expressed in vestibular HCs, such as *Rfx2*, *Rfx5* or *Rfx7*. The absence of abnormalities of ciliogenesis or planar cell polarity in the mutant mice, coupled with the timing of HC death, only after P12, suggest a novel role for RFX TFs in hearing, independent of ciliogenesis. The exact mechanism of OHC degeneration in *Rfx1/3* cKO mice is yet unclear, but the loss of outer but not inner HCs could result from the well-known greater susceptibility OHCs to cochlear insults or from compensatory effects of other *Rfx* genes. In addition, differential requirement of TF for HC survival has been previously described; For example, while *Barhl1* is expressed in both auditory and vestibular HCs, it is only required for the survival of cochlear HCs^41^. Likewise, while *Gfi1* is expressed in all cochlear HCs, OHCs show a greater dependence on its expression compared with IHCs^7^,^42^.

Our data show that *Rfx1/3* are necessary for OHC survival at the latest stages of their development. In the absence of *Rfx1/3*, OHCs undergo very fast programmed cell death. The timing of the OHC death in the *Rfx1/3* cKO mice coincides with two critical processes both related to RFX biology. First, the kinocilia, which are normally resorbed in wild-type HCs during this period, are retained in the mutant mice. This suggests a largely unappreciated but possible link between normal recess of kinocilia and HC survival. Second, the endocochlear potential is also developed exactly at this developmental stage^43^. The appearance of endocochlear potential should dramatically increase the resting current through the mechanotransduction channels, Ca^2+^ influx in the HCs through these channels and associated metabolic stress in the OHCs^44^. Metabolic stress after noise exposure has been shown to result in DNA damage^45^. Interestingly, RFXs have a known role in cellular homeostasis through regulation of DNA integrity^46^,^47^. Therefore, it is possible that RFX may regulate HC survival also through maintenance of DNA integrity.

Perhaps the most significant contribution of this study is in the identification of RFX as a new family of TFs with a role in regulating the HC transcriptomes. Previously described transcription factors important for HC differentiation such as ATOH1 (ref. 5), POU4F3 (refs 8, 42, 48) and GFI1 (refs 7, 42) are necessary for early (embryonic) HC differentiation and survival, while RFX1/3 are crucial for the latest stage of HC development. In *Drosophila*, rfx is directly regulated by atonal (the Atoh1 homologue), and in conjunction with a forkhead transcription factor, fd3F, regulates the differentiation of the chordotonal (mechanosensory) neurons involved in hearing in Drosophila^49^,^50^. In mouse, the closest orthologue to fd3F is *Foxj1*, which is also expressed in the auditory and vestibular HCs (Supplementary Data 2), and could be required together with RFX to regulate the maturation of HCs. Interestingly, a recent manuscript by Costa *et al*.^9^ showed that forced expression of ATOH1, POU4F3 and GFI1 in stem cells results in immature HC like-cells, which specifically lack the expression of the orthologues of some of the target genes of fd3F, consistent with our hypothesis that the RFX pathway is necessary for HC terminal differentiation. Future studies will explore the RFX-specific transcriptional cascade in the developing HCs and determine whether this family of TF can be applied for regenerative treatments for hearing loss.

## Methods

### Animals

Wild-type ICR mice were obtained as time-mated animals from Charles River Laboratories (Maryland). The *Atoh1/nGFP* mice^51^ were kindly provided by J. Johnson and maintained by intercrossing the transgenic mice. Zebrafish stocks were maintained at 28.5 °C. Embryos were generated from natural matings of *ppv3b:GFP* adult zebrafish (kindly received from B. McDermott, Case Western Reserve University). Embryos were cultured in embryo medium^52^ at 28.5 °C until harvesting at 5 dpf. All procedures involving animals were carried out in accordance with the National institutes of Health Guide for the Care and Use of Laboratory Animals and have been approved by the Institutional Animal Care and Use Committee at the University of Maryland, Baltimore (protocol numbers 1112005 and 0313013).

### Generation of conditional KO mice

Generation of *Rfx3*^*flox/flox*^ mice is described in Bonnafe *et al*.^53^. *Rfx1*^*flox/flox*^ were generated as follows: the targeting vector contained two loxP sites (P) inserted into the introns flanking exon 10 of the *Rfx1* gene, and an frt (F) flanked neomycin resistance (pgk-neo-pA) cassette in the intron downstream of exon 10 (Supplementary Fig. 12). Exon 10 contains an essential portion of the *Rfx1* DNA-binding domain. The linearized targeting vector was transfected into embryonic stem (ES) cells by electroporation and G418-resistant clones were selected. Screening for homologous recombination was done by PCR using an internal primer and a primer situated upstream of the 5′ end of the targeting construct. Positive clones were confirmed by Southern blot analysis using 5′, 3′ and internal probes. The neo resistance gene was excised in recombinant ES cell clones by transfection with the pCAGGS-Flpe vector and selection with puromycin. Clones were isolated and screened for G418 sensitivity. Excision of the neo resistance gene was controlled by Southern blot analysis with an internal probe. Information on primers, restriction sites and probes will be made available on request. Correctly targeted ES cell clones were injected into C57BL/6J blastocysts and implanted into recipient females. Germ line transmission of the recombinant *Rfx1* allele was confirmed by PCR and Southern blotting. Conditional deletion of exon 10 was achieved by breeding in a *Gfi1-Cre* transgene. After deletion of exon 10, splicing of exon 9 to exon 11 leads to a frame shift. For generation of mice with a conditional deletion of *Rfx1* or *Rfx3*, *Rfx1*^*flox/flox*^ and *Rfx3*^*flox/flox*^ mice were crossed with *Gfi1-Cre* knock-in mice^54^. The *Gfi1-Cre* mice express Cre-recombinase in the majority (>90%) of the inner ear HCs. For the generation of a mouse line homozygous for both *Rfx1*^*flox/flox*^ and *Rfx3*^*flox/flox*^ recombinant alleles, *Rfx1*^*flox/flox*^ and *Rfx3*^*flox/flox*^ mice were crossed and the progeny were intercrossed. For the generation of mice with a conditional deletion of *Rfx1* and *Rfx3* in HCs of the inner ear, *Rfx1*^*flox/flox*^*;Rfx3*^*flox/flox*^ mice were crossed with *Gfi1-Cre* knock-in mice and the progeny were intercrossed until obtaining *Rfx1*^*flox/flox*^*;Rfx3*^*flox/flox*^*;Gfi1*^*Cre/+*^ mice.

### Genotyping

Genotyping was performed by PCR on genomic DNA extracted from tail tips using the Extract-N-Amp Tissue PCR Kit (Sigma-Aldrich) following the manufacturer’s instructions. Primer sequences were as follows: genotyping of *Rfx1* mice forward (Fwd) (5′-GCAGGTGGCTAGTAGCAAGT-3′) and reverse (Rev) (5′-AGAGCTGAGCAAGGGAGTTA-3′). PCR amplification results in the following band sizes: wild type 709 bp, floxed allele 817 bp, excised allele 500 bp; Genotyping of the *Rfx3* mice: Fwd (5′-GTCATGCTGGAAAATTTGAAG-3′) and Rev (5′-AGTTGGCTTCTAACTTCTATG-3′). PCR amplification results in the following band sizes: wild type 592 bp, floxed allele 800 bp, excised allele 300 bp; Genotyping of the *Gfi1* mice: Fwd (5′-GGGATAACGGACCAGTTG-3′), Rev-WT (5′-CCGAGGGGCGTTAGGATA-3′) and Rev-Cre (5′-GCCCAAATGTTGCTGGATAGT-3′). PCR amplification results in the following band sizes: wild type 609 bp, Cre allele 672 bp^54^.

### Cell separation by flow cytometry

Newborn (P1) *Atoh1/nGFP* mice were used to separate the inner ear cells to HCs, ENHCs and NECs. Inner ears from four to eight mice were used for each biological replicate. Tissue harvesting and processing of mouse inner ears for flow cytometry was performed as previously described without modifications^10^. The auditory sensory epithelia and vestibular tissues were collected in 48-well plates containing 0.5 mg ml^−1^ thermolysin (Sigma) and incubated for 20 min in a 37 °C/5%CO2 humidified tissue culture incubator to allow partial digestion of the extracellular matrix. The thermolysin was then aspirated, replaced with Accutase enzyme cell detachment medium (eBioscience) and tissues were incubated for 3 min in a tissue culture incubator followed by mechanical disruption using a 23G blunt ended needle connected to a 1 ml syringe. The 3-min incubation and mechanical disruption were performed twice. The reaction was stopped by adding an equal volume of IMDM supplemented with 10% heat-inactivated foetal bovine serum (FBS). After confirming cellular dissociation by direct visualization using an inverted tissue culture microscope, cells were passed through a 40-μm cell strainer (BD) to remove cell clumps. Following dissociation, we stained the cells with CD326-APC (1:2,000, Biolegend) diluted in IMDM-10% FBS for 30 min on ice. CD326 is a marker for the epithelial cells in the inner ear^10^. Cells that were positive both for GFP and CD326 are HCs, positive for CD326 and negative for GFP are ENHCs, and negative both for GFP and CD326 are NECs. Of note, possible minor expression of GFP in neuronal cells^55^ was eliminated from the sorted cells by restricting HCs to cells that are both GFP and CD326 positive. Pillar cell expression of GFP was not strongly visualized in our prep as demonstrated in Fig. 1a. For cell separation from *ppv3b:GFP z*ebrafish, embryos were dissociated as follows (previously reported^56^): Five dpf larvae were dissociated by incubation in trypsin (Trypsin-EDTA 0.25%, Life Technologies) for 30 min with intermittent mechanical disruption by trituration. The reaction was stopped by adding HBSS supplemented with 10% FBS and 100 μg ml^−1^ DNaseI. Cells were filtered through a 70-μm cell strainer (BD Biosciences), centrifuged at 800*g* for 7 min at 4 °C and resuspended in HBSS supplemented with 10% serum. Following each sort, a small aliquot of cells was re-analysed to determine cell purity. All cell sorting experiments were performed using a Beckman Coulter MoFlo XDB flow cytometer and cell sorter (Beckman Coulter Genomics, Danvers, MA, USA) or BD FACSARIA Cell Sorter (BD Biosciences) at the University of Maryland School of Medicine Greenebaum Cancer Center Flow Cytometry Shared Services.

### RNA extraction

For the samples processed by RNA-seq, TRIzol LS Reagent (Life Technologies) was added to the sorted cells (3 vol of reagent per 1 vol of sample) immediately following sorting after the completion of the post sort analysis. Total RNA was extracted from sorted cells using the Direct-zol RNA MiniPrep kit (Zymo Research). RNA integrity was evaluated with a RNA Pico Chip on a 2100 Bioanalyser (Agilent). For the samples processed by microarrays, RNA was extracted using the RNeasy Plus Micro Kit (Qiagen). All samples used for gene expression analysis had a RNA integrity score >8.5.

### Gene expression profiling and analysis

*Microarrays data analysis*. Expression profiles were recorded from six cellular populations (three cell-types: HCs, ENHCs and NECs, isolated from two inner ear organs: the cochlea and the vestibular system). These six conditions were probed in biologically independent triplicates using Illumina MouseRef-6 v2.0 Expression BeadChips (Illumina, San Diego, CA, USA). These arrays contain >45,000 probes which collectively interrogate all RefSeq annotated mouse genes (>26,000 genes) and ∼7,000 RefSeq predicted genes. Expression levels were calculated using Illumina's Bead-Studio package. Probes not readily detected in the data set were filtered out using detection *P* values assigned by Bead-Studio to each measurement and requiring that each probe be detected (*P* value<0.01) in at least two samples. This criterion left 23,051 probes, corresponding to 17,275 annotated and predicted genes for subsequent analyses. Arrays were then normalized using quantile normalization. Hierarchical clustering, using average-linkage method, was applied to assess overall similarity between the different conditions. Differentially expressed genes in the data set were identified using two-way analysis of variance (ANOVA) analysis. 6,556 probes were significantly (false discovery rate <5%) associated with either factor (cell-type or organ) or showed significant interaction between these two factors (that is, different cell-type expression patterns between the two organs). Main expression patterns exhibited by the set of differentially expressed probes were delineated by cluster analysis using the CLICK algorithm implemented in the EXPANDER package^57^. Prior to clustering, expression levels of each probe were standardized to mean=0 and s.d.=1. To define cell type-specific marker genes, we first examined the microarray data set for cutoff values that correspond with ‘expression’ compared with ‘no-expression’ of probes, and observed that that all probes flagged as not expressed (that is, detection *P* value>0.05) had expression levels <120 (a.u.), while all the probes flagged as expressed (detection *P* value<0.01) had expression levels >125. We next set the following stringent criterion—for a gene to be identified as a ‘marker gene’ it must be expressed at a value >250 in the cell type of interest and with a value of <120 in all other samples.

*RNA-seq*. To generate the mouse HC versus ENHC libraries, 5–20 ng of total RNA were used as input for the NuGEN Ovation RNA-seq V2 System. Amplifications yield 4–5 μg SPIA complementary DNA (cDNA). About 100 ng of fragmented cDNA was used as an input for the Ovation Ultralow Library System. For the zebrafish RNA-seq, 70 ng of total RNA was used as starting material without the need for pre-amplification. Specifically, Illumina RNAseq libraries were prepared with the TruSeq RNA Sample Prep kit (Illumina) per manufacturer’s protocol. Adapters containing 6-nt indexes were ligated to the double-stranded cDNA. The DNA was purified between enzymatic reactions and the size selection of the library was performed with AMPure XP beads (Beckman Coulter Genomics). Libraries were pooled, and each received 0.25 a lane of sequencing on an Illumina HiSeq 2500 with a paired-end 100 base configuration. Library construction and sequencing were performed at the Institute for Genome Sciences of the University of Maryland, School of Medicine (IGS).

*RNA-seq data analysis*. Sequenced reads were aligned to the mouse and zebrafish genomes (mm9 and Zv9) using TopHat^58^. Based on profiles of base-calls quality, the reads in the mouse data set were trimmed and only the first 50 nt were used for the alignment. Gene expression levels (read counts) were calculated using HTseq^59^ based on ENSEMBL gene annotations^60^. The mouse and zebrafish data sets were then normalized using quantile normalization and then a floor value of 20 reads was set (to avoid inflation of fold-change estimates due to division by low counts). In the mouse and zebrafish data sets, 12,814 and 17,668 genes were detected as expressed, respectively.

*GO analysis*. Enriched Gene Ontology (GO) categories in the clusters were detected by DAVID^61^ using the set of all genes expressed in the data set as the background set.

*Analysis of cis-regulatory motifs*. Over-represented sequence motifs in promoters were searched using AMADEUS^62^. The set of promoters of all genes detected as expressed in each analysed data set was used as the background set. The promoter regions examined spanned from 1,000 nt upstream to 500 nt downstream the TSS. Mouse, human and zebrafish orthologous genes were mapped using ENSMART utility^60^. In all tests of HC-elevated genes, the top scoring motif matched the known binding signature of RFX TFs. The location distribution of this motif consistently showed a very sharp peak at the TSS (Supplementary Fig. 6b), indicating that RFX commonly regulates the expression of its target genes by binding to their very proximal promoter region. Accordingly, the enrichment was most statistically significant when the search was narrowed to the interval between 200 nt upstream and 100 nt downstream of the TSSs. The results reported correspond to this interval.

*Deafness-related loci*. A list of cloned human deafness-related genes and deafness loci for which the underlying gene was not cloned yet was compiled from the Hereditary Hearing loss Homepage^63^. Human genes located within deafness loci were extracted using a Perl script. Mouse homologues of these genes were found using NCBI's HomoloGene^64^.

### ChIP-seq

ChIP was performed as previously described^65^: MIN6 beta cells were crosslinked with 1% formaldehyde for 10 min at room temperature. The reaction was stopped by adding of glycine to 0.125 M followed by cold PBS washes. Cells were lysed in TE buffer supplemented with protease inhibitors and 0.5% NP-40. Following centrifugation, the nuclei were lysed in TE supplemented with 0.5 M NaCl, 1% Triton X-100, 0.5% sodium deoxycholate and 0.5% sarcosyl. Crosslinked chromatin was resuspended in TE plus 100 mM NaCl, sheared by sonication and cleared by two cycles of centrifugation at 15,000*g* for 15 min. About 10 μg of the resulting sheared chromatin was diluted 10-fold with IP buffer (20 mM Tris-HCl, pH 8.0, 200 mM NaCl, 2 mM EDTA, 1 mM phenyl-methyl-sulfonyl-fluoride, 0.1% sodium deoxycholate, 0.1% SDS) containing 50 μg ml^−1^ salmon sperm DNA, 100 μg ml^−1^ Escherichia coli tRNA and 1 mg ml^−1^ bovine serum albumin. Diluted chromatin was then incubated at room temperature with protein A-Sepharose beads, polyclonal anti-RFX1 and anti-RFX3 sera^37^. Beads were washed twice with IP buffer, twice with IP buffer containing 500 mM NaCl, twice with 20 mM Tris-HCl, pH 8.0, 0.25 M LiCl, 2 mM EDTA, 0.5% NP-40, 0.5% sodium deoxycholate and once with TE containing 0.1% NP-40. Chromatin fragments bound to the beads were eluted by incubation in 100 mM Tris-HCl, pH 8.0, 1% SDS for 10 min at 65 °C. Eluted chromatin was digested for 1 h at 37 °C with 400 mM NaCl, 200 μg ml^−1^ proteinase K, followed by incubation at 65 °C overnight to reverse the crosslinks. DNA was extracted with phenol-chloroform, resuspended in TE and quantified with a Qubit Fluorometer from Invitrogen. To generate the ChIP-sequencing library, 10 ng of DNA were used as input for the ChIP-seq Sample Preparation Kit (Illumina). Sequencing was performed with the Genome Analyser II (Illumina) using the 36 Cycle Sequencing Kit v2. Sequences were derived from a single channel of the flow cell. Data were processed using the Illumina Pipeline Software package v1.0.

### Immunohistochemistry on paraffin sections

Immunohistochemistry reactions were performed on 5-μm sections of paraffin-embedded inner ears from P1 wild-type ICR mice. Sections were deparaffinized with xylene and rehydrated with a decreasing ethanol gradient, followed by antigen retrieval with sodium citrate buffer (pH6). Sections were blocked with PBS supplemented with 0.2% Tween-20, 10% normal goat serum and 1% bovine serum albumin for 30 min at room temperature before incubation with rabbit polyclonal anti-RFX3 (HPA035689, Sigma-Aldrich, 1:200) overnight at 4 °C. Antibody labelling was performed with a biotin-conjugated anti-rabbit IgG (1:500, Life Technologies) and detection with Alexa Fluor 594-conjugated streptavidin (1:500, Life Technologies) for 1 h at room temperature. The nuclei were counterstained with 300 nM of DAPI (Life Technologies). Samples were then mounted in anti-fade medium (ProLong Gold antifade reagent, Life Technologies). Images were acquired with a Nikon Eclipse E800 coupled with a Qimaging Retiga EXi Fast1394 and analysed with the Volocity Image Analysis Software (Perkin Elmer).

### Immunohistochemistry on whole mounted samples

Sensory epithelia from cochlear and vestibular systems were dissected from mice at the ages specified in the figure legends. Tissues were permeabilized in PBS-0.5% tween20, blocked in M.O.M. Mouse Ig Blocking Reagent (Vector Laboratories) for 1 h at room temperature and incubated with mouse monoclonal Anti-Acetylated Tubulin (Sigma) diluted in M.O.M. Diluent at 1:500 overnight at 4 °C or blocked in PBS-0.2% tween20-10% Donkey serum and incubated with a goat anti-Prestin antibody (1:200, SC22692, Santa Cruz Biotechnology). Detection was performed with an Alexa Fluor 546 conjugated anti-mouse IgG (1:500, Life Technologies) or a Alexa Fluor 546 conjugated anti-goat IgG (1:500, Life Technologies) supplemented with Alexa Fluor 488 conjugated Phalloidin (1:500, Life Technologies) and 300 nM of DAPI for 1 h at room temperature. Samples were then mounted in anti-fade medium (ProLong Gold antifade reagent). Images were acquired with a Zeiss LSM 510 confocal microscope. For quantification of planar cell polarity, sensory epithelia from 3-day-old mice were obtained and labelled for acetylated tubulin as described, the orientation of 20 cells from the middle and basal turns of each cochlea (consisting of five HCs from each row) was measured using the ImageJ software (http://imagej.nih.gov/ij/) as follows: a line perpendicular to the row of pillar cells and extending through the HCs was drawn, as well as a line going from the centre of each HC to their kinocilium. The orientation of the bundle is calculated by measuring the angle obtained at the intersection of these two lines. Graphs were plotted using GraphPad Prism and statistical analyses were performed using a one way ANOVA, post test: Tukey’s test.

### FM1-43 staining

Sensory epithelia from cochleae were dissected from P1 and P3 mice and cultured for 24 h on CellTak-treated glass bottom culture dish in DMEM/F12 (Life Technologies) supplemented with 10% FBS and 10 μg ml^−1^ ampicillin. Culture dishes with adherent cochlear explants were rinsed once with HBSS supplemented with 10 mM HEPES pH 7.2 (HHBSS), followed by incubation with 3 μM FM1-43 (Life Technologies) for 10 s, immediately followed by four washes with HHBSS. Images were acquired with a Zeiss LSM 510 confocal microscope.

### Scanning electron microscopy

The temporal bones from 8-, 15-, 22- and 90-day-old mice were dissected in ice-cold Leibowitz L-15. Cochleae were exposed and small holes were made at the apex and the base to allow fixative penetration. Cochleae were fixed in 2.5% glutaraldehyde in 0.1 M cacodylate buffer supplemented with 2 mM CaCl_2_ for 1.5 h at room temperature or at 4 °C overnight. Then, the cochlear partition was dissected in an ice-cold PBS or distilled water. Dissected specimens were dehydrated in a series of ethanol, followed by critical point drying from liquid CO_2_, and sputter coated with platinum (5.0 nm, controlled by a film-thickness monitor) and observed with a field-emission electron column of Helios 660 NanoLab instrument (FEI Co., Hillsboro, OR). Alternatively, a modified osmium–thiocarbohydrazide–osmium procedure was used. Briefly, the fixed and dissected specimens were treated with 1% osmium tetroxide three times with a 30-min water wash and a 20-min incubation in saturated thiocarbohydrazide between each osmium tetroxide treatment. Then, the specimens were dehydrated as described above and imaged with Quanta 200 SEM (FEI).

### Auditory physiology

Auditory brainstem response (ABR) and distortion product otoacoustic emissions (DPOAE) were completed using methods reported previously (full details below)^66^,^67^,^68^,^69^. Mice were anesthetized with a ketamine/xylazine (18:2 mg ml^−1^) solution (5–9 μl g^−1^ body weight). The following genotypes were tested at P22: *Rfx1/3* cKO (*n*=4), wild type (*n*=10); and at P90: *Rfx1* cKO (*n*=3), *Rfx3* cKO (*n*=8), *Rfx1/3* cKO (*n*=10), wild type (*n*=6). All mice in each age group were littermates and consisted of all males and females born in the litter.

*Auditory brainstem response*. Tone burst stimuli at 8, 16 and 32 kHz were presented via high frequency transducers coupled at the ear via polyethylene tubing tubing. Subcutaneous electrodes were placed at the nuchal crest, posterior to the left pinna and at the hip for noninverting, inverting and ground leads. Traditional signal averaging was used where ongoing electroencephalographic activity was amplified (200,000 × ), filtered (300 to 3,000 Hz), digitized (100 kHz) and averaged (256 to 512 averages per waveform). ABR intensity series was collected with a descending series of stimulus levels (5-dB steps) beginning at 110 dB peSPL. ABR thresholds were determined and compared between knockout and controls using ANOVA.

*DPOAE*. DPOAE frequencies (f1, f2, f2/f1 ratio=1.25) at equal levels (L1=L2=60 dBSPL), 150 ms duration, were generated and routed through separate drivers to mix acoustically in the ear canal. Geometric mean ((f1 × f2)^0.5^) frequencies ranged from 6.0 to 48.5 kHz (eight frequencies per octave). Ear canal sound pressure levels were recorded with a calibrated low noise probe microphone. Microphone output was amplified, sampled (200 kHz) and subjected to fast Fourier transform (FFT). The amplitude of f1, f2 and the 2f1–f2 distortion products were measured from the FFT waveform. The noise floor was measured from the amplitudes in the five frequency bins above and below the 2f1–f2 component. The mean DPOAE amplitude across all tested primary frequency pairs was calculated and compared between knockouts and wild-type controls using ANOVA.

### Western blot analysis

Cochlear ducts were dissected from P12 and P15 wild-type and *Rfx1/3* cKO mice and flash frozen in dry ice. Each biological replicate consisted of tissue collected from four ears. Two biological replicates (a total of eight ears) were collected for analysis from each time point and genotype. Cochlear tissues were subjected to lysis in RIPA buffer (Pierce). Total protein was quantified (Bio-Rad) and normalized for protein levels. Proteins were separated by SDS–PAGE electrophoresis using 12% precast Nupage Bis/Tris gels under reducing conditions using MES running buffer (Invitrogen) and transferred onto a nitrocellulose membrane (Amersham Biosciences) using a semidry transfer apparatus (Bio-Rad) and Nupage-MOPS transfer buffer. For immunoblot analysis, membranes were blocked with TBS-Tween containing 5% dry milk and then were incubated with a mouse IgG against human caspase 3 (1:200, Santa Cruz Biotechnology), a mouse IgG against human BAX (1:150, Santa Cruz Biotechnology), a rabbit IgG-HRP against human Actin (1:400, Santa Cruz Biotechnology) and a rabbit IgG against human BCL2 (1:200, Santa Cruz Biotechnology). Bound antibodies were detected by secondary antibodies conjugated to horseradish peroxidase (Santa Cruz Biotechnology). Signal detection was performed by an enhanced chemiluminescence (ECL) detection reagent (Life Technologies). Approximate molecular masses were determined by comparison with the migration of prestained protein standards (Bio-Rad).

## Additional information

**Accession codes:** All gene expression data were deposited in the Gene Expression Omnibus under accession codes GSE64543 for the microarray and RNA-seq data and GSE72272 for the ChIP-seq data.

**How to cite this article:** Elkon, R. *et al*. RFX transcription factors are essential for hearing in mice. *Nat. Commun.* 6:8549 doi: 10.1038/ncomms9549 (2015).

## Supplementary Material

Supplementary Information Supplementary Figures 1-13 and Supplementary References.

Supplementary Data 1 Gene expression profiles in the inner ear measured using microarrays. Expression profiles were recorded from six cellular populations (three cell-types: HCs, ENHCs and NECs, isolated from two inner ear organs: the cochlea and the vestibular system) using independent biological triplicates. This dataset contains quantile-normalized expression levels for microarray probes that were flagged as expressed in at least two samples. Assignment of probes to the gene clusters shown in Figure 1 is indicated in column V.

Supplementary Data 2 Gene expression profiles in the inner ear measured using RNA-seq. Gene expression was recorded in hair-cells (HC) and non-HCs populations. This dataset contains expression levels for genes covered by at least 20 reads. Expression levels were normalized by quantile normalization and a floor value of 20 was set. Assignment of genes to the clusters shown in supplementary Figure 4 is indicated in column K.

Supplementary Data 3 Assignment of the mouse orthologs of known human deafness-causing genes to gene expression clusters.

Supplementary Data 4 Marker genes of inner-ear HCs.

Supplementary Data 5 Putative HC-enriched RFX target genes.

Supplementary Data 6 Gene expression profiles in Zebrafish hair cells. Expression levels measured using RNA-seq in transgenic zebrafish which express GFP predominantly in hair cells. Data were recorded for sorted GFP-positive (+) and negative (−) cells from 5 day post fertilization (dpf) larvae using flow cytometry.
