# Supplementary material for: RFX transcription factors are essential for hearing in mice
Source: Nat Commun. 2015 Oct 15;6:8549. doi: 10.1038/ncomms9549 (PMC4634137; doi:10.1038/ncomms9549)
Supplement: Supplementary Information — Supplementary Figures 1-13 and Supplementary References. [file ncomms9549-s1.pdf]

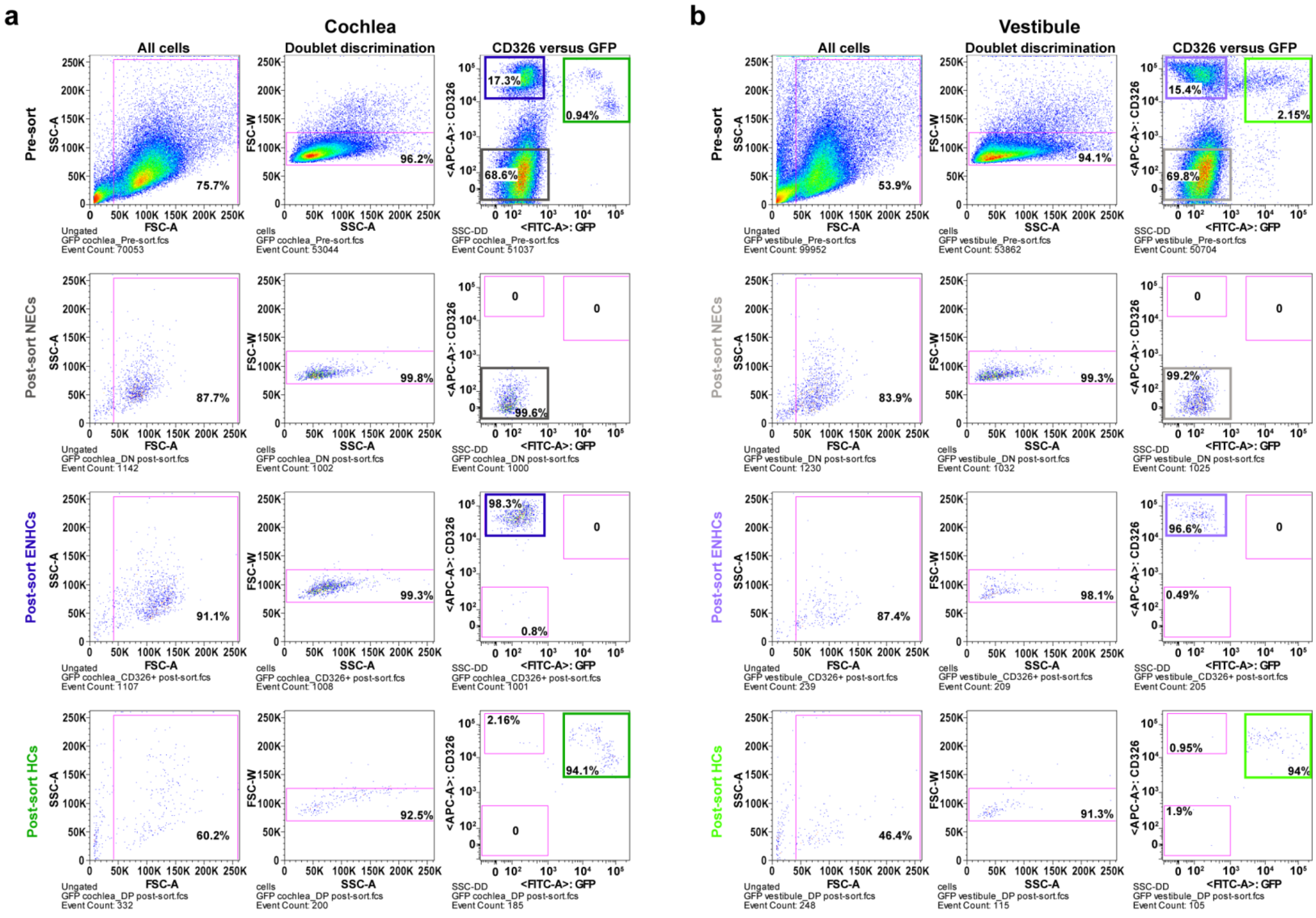

Supplementary Figure 1. Flow cytometry and sorting of inner ear HCs, ENHCs, and NECs. Cochlear (**a**) and vestibular (**b**) cells from the *Atoh1/nGFP* mice were dissociated and stained with CD326 that marks epithelial cells. Each panel shows a dot plot of granularity (SSC-A, Y-axis) versus size (FSC, X-axis) in the left figure; Width (FSC-W, Y-axis) versus granularity (SSC-A) to exclude cell doublets and aggregates in the middle figure; and CD326 (Y-axis) versus GFP (X-axis) expression in the right figure. Pre-sort panels indicate the distribution of the cells prior to sorting. Purple boxes mark the sorting gates. Post-sort panels show the cellular characteristics post sorting. All sorted populations were at least 94% pure.

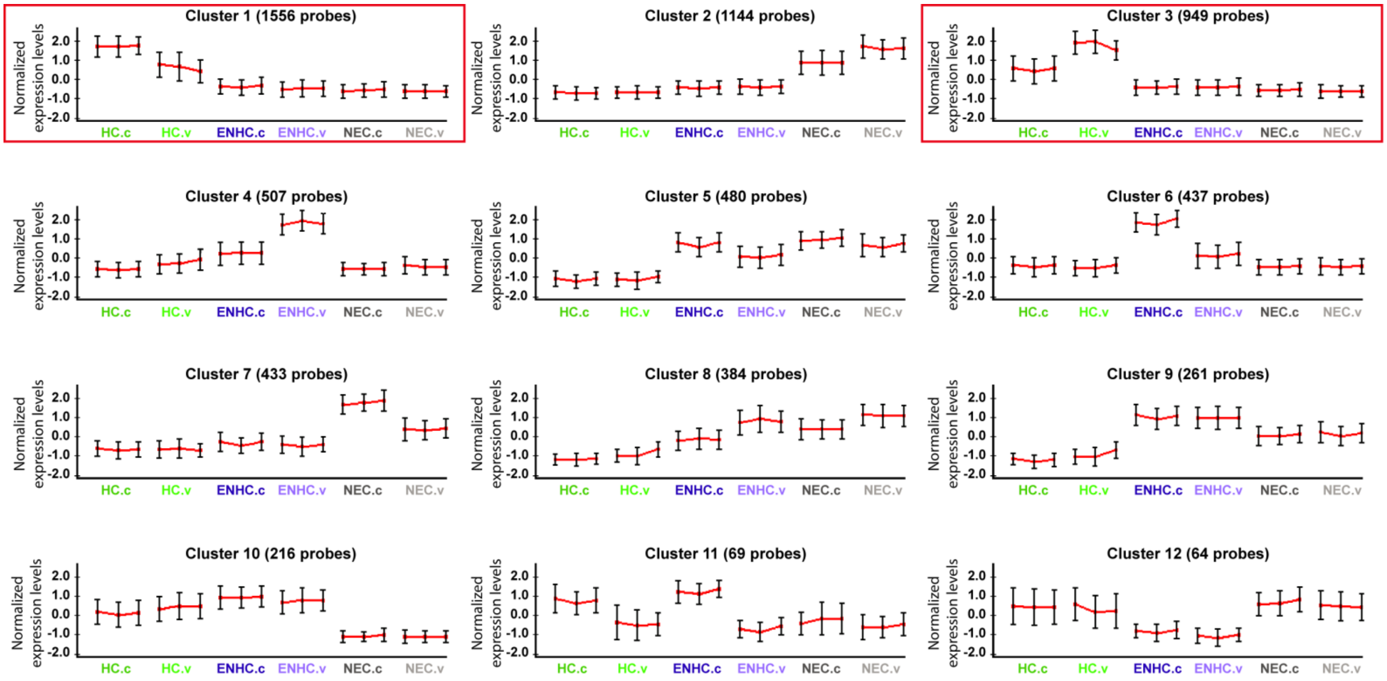

Supplementary Figure 2. Cluster analysis of inner ear gene expression. Cluster analysis applied to the set of differentially expressed genes detected twelve main patterns of gene expression. Two of the clusters (marked with a red box) contained genes with an elevated expression in HCs compared with ENHCs and NECs. Each cluster is represented by the mean pattern of its gene expression. Expression levels of each gene were standardized (mean=0, SD=1) prior to clustering, to cluster together genes that share the same pattern, but not necessarily the same magnitude of expression. Measurements were done on biologically independent triplicates. Clusters are numbered according to the number of genes they contain. Data represent results from three biological replicates. Error-bars: +/- SD.

a

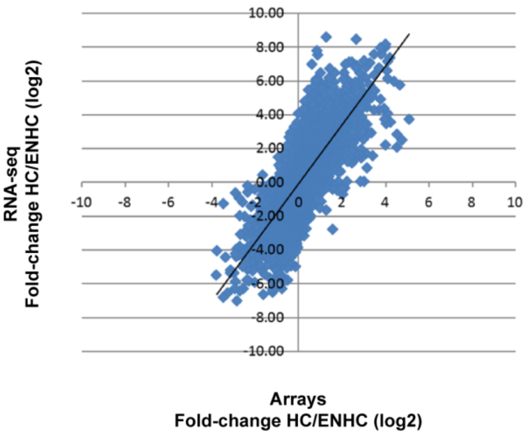

b

		RNA-seq			Microarray		
**Gene id**	**Symbol**	**HC**	**ENHC**	**FC**	**HC**	**ENHC**	**FC**
**83762**	***Otof***	1565.5	59.3	26.4	617.9	90.0	6.9
**17921**	***Myo7a***	1329.8	27.5	48.4	196.5	89.4	2.2
**14581**	***Gfi1***	626.0	22.5	27.8	1847.2	110.7	16.7

Supplementary Figure 3. Correlation between microarray and RNA-seq measurements. HC and ENHC RNA was extracted from newborn *Atoh1/nGFP* mice and gene expression levels were measured using microarrays and RNA-seq. **a.** The relative expression in HCs versus ENHCs was calculated for each gene in the microarray and RNA-seq datasets (microarray – X axis; RNA-seq – Y axis; both axes are in log2). Fold-change measurements were significantly correlated between the two techniques (correlation coefficient = 0.7). The plot also demonstrates the increased dynamic range of the RNA-seq measurements (up to 64- versus 8-fold difference in expression levels). **b.** Examples of genes encoding HC-enriched transcripts as detected by both RNA-seq and microarrays, showing the improved dynamic range of RNA-seq compared to microarrays; the fold of enrichment (fold-change; FC) measured by RNA-seq is markedly higher than the ones measured by microarrays. This improvement is mainly due to the fact that while the analog signal measurement of microarray has relatively high background noise, the digital measurement of RNA-seq has essentially no background noise.

a

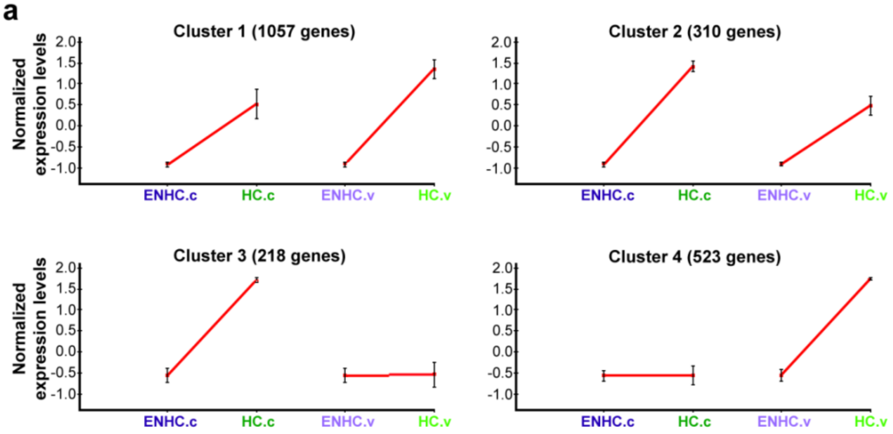

b

Cluster	Enriched GO category	# of genes	FDR
Cluster 1	Cilium	28	1.45E-08
Cluster 1	Cytoskeleton	98	1.91E-06
Cluster 1	Sensory perception	33	4.35E-06
Cluster 2	Cation channel activity	13	0.016
Cluster 2	Neurological system process	22	0.025

c

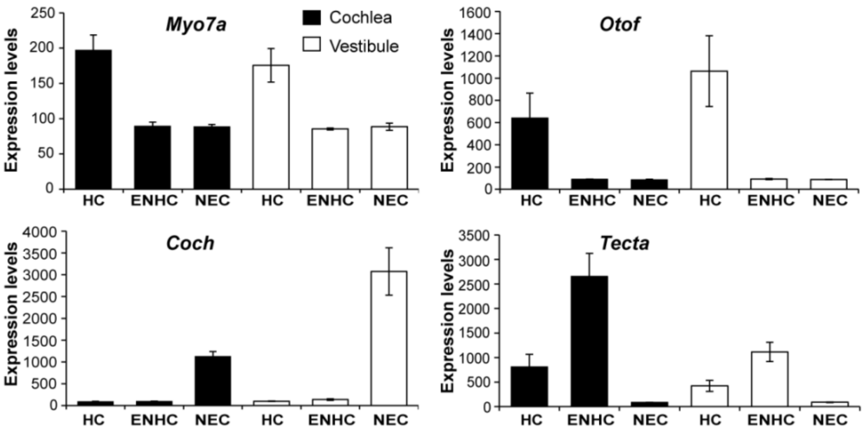

Supplementary Figure 4. Gene expression analysis of HCs and ENHCs using RNA-seq. a. Results of cluster analysis applied to the genes that were detected as differentially expressed in the HC/ENHC RNA-seq dataset. Clusters 1 and 2 represent genes with an increased expression in HCs compared with ENHCs in both the auditory and the vestibular systems. Cluster 1 shows a mean pattern of higher enrichment in the vestibular HCs compared with the cochlear HCs, and in cluster 2 this pattern is reversed. Cluster 3 and 4 represents genes with a uniquely enriched expression in cochlear or vestibular HCs, respectively. **b.** Enriched GO categories in clusters 1 and 2. **c.** Expression patterns of selected deafness-causing genes with a differential pattern of expression. Overall, deafness-causing genes were statistically over-represented in the clusters of genes whose expression was HC-enriched, but we also detected deafness-causing genes that showed a different expression pattern in the inner ear. For example, *Myo7a* is elevated in HCs; *Otof* is HC-enriched with a higher level in the vestibular HCs; *Coch* is enriched in the NECs and *Tecta* is enriched in ENHCs. Expression data are based on the microarray dataset (n=3 biological replicates; error bars: +/- SD).

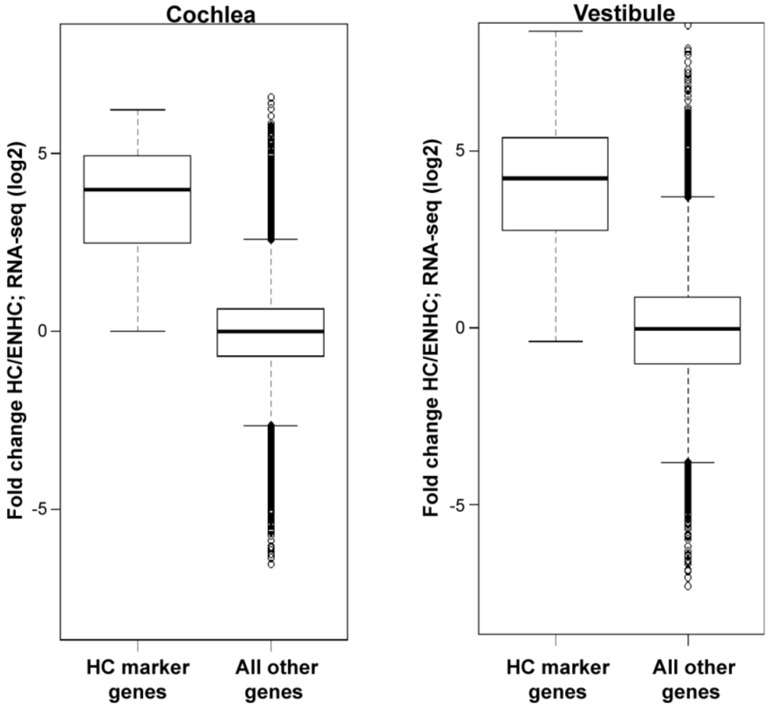

Supplementary Figure 5. RNA-seq confirmation of HC-marker genes. HC-marker genes as defined based on the microarray data were confirmed using RNA-seq measurement of gene expression in an independent set of samples. Ninety five percent of the HC-marker genes showed more than 2-fold expression enrichment in HCs compared to ENHCs, demonstrating high specificity. Because RNA-seq has greater sensitivity than microarray, especially for detecting differential expression of lowly expressed genes, there are many more HC-marker genes to be detected. In addition, since we set a stringent criterion for calling HC-marker genes, there are many genes whose expression is enriched in HCs that were not called as marker genes. Boxes indicate the 1st and 3rd quartiles; the horizontal band inside the box indicates the median. The whiskers extend to the most extreme data point, which is no more than 1.5-times the interquartile range from the box.

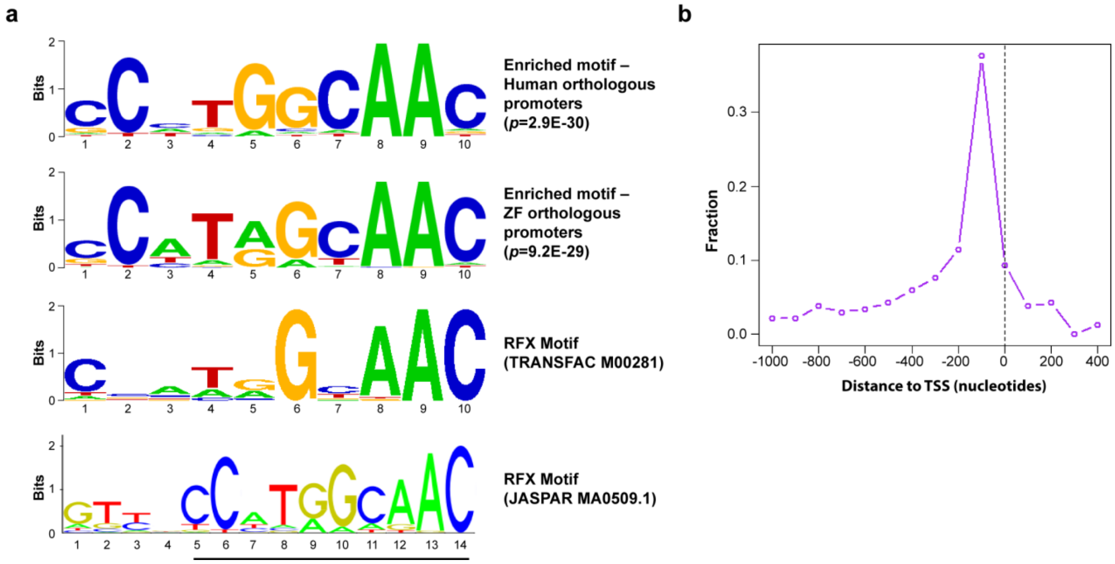

Supplementary Figure 6. Enrichment of the RFX motif in promoters of HC genes is evolutionarily conserved. a. *De-novo* motif analysis revealed that the promoters of the human and zebrafish orthologous genes of the murine genes whose expression was elevated in inner ear HCs, as detected by our microarray and RNA-seq analyses, are significantly enriched too for the RFX binding signature (enrichment *p*-value=2.9E-30 and 9.2E-29 for the human and zebrafish promoters, respectively, hypergeometric test). Of note, the motif detected by our computational analysis matches the RFX motif recorded in TRANSFAC DB (signature length is 10 bp). JASPAR DB reports an extended motif for RFX whose length is 14 bp (a palindromic motif of 6 nt with a spacer of 2 nt)[1]. The computationally detected motif corresponds to the most informative part of the motif reported by JASPAR. **b.** Location distribution of the computationally detected RFX motifs showed very sharp peak near the transcription start site (TSS) of the target genes. The distribution shown here corresponds to occurrences of the RFX motif detected in the mouse promoters of the genes encoding HC-enriched transcripts.

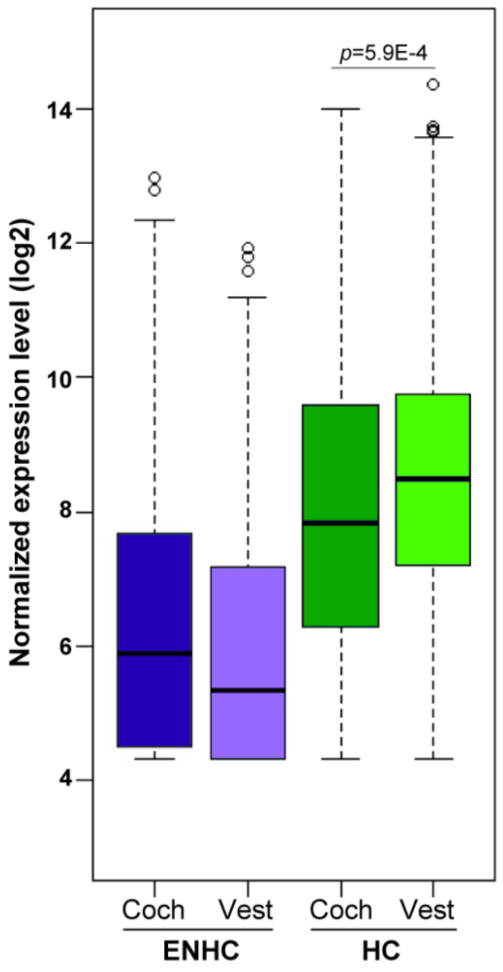

Supplementary Figure 7. Expression of RFX target genes at P1 is higher in vestibular HCs than cochlear HCs. The expression of the set of 225 predicted RFX HC target genes is significantly higher in vestibular HCs than cochlear HCs (*p*-value=5.9E-4; Wilcoxon's test). As RFX factors are required for late stages of differentiation this result might reflect the fact that the vestibular system precedes auditory differentiation. Boxes indicate the 1st and 3rd quartiles; the horizontal band inside the box indicates the median. The whiskers extend to the most extreme data point, which is no more than 1.5-times the interquartile range from the box.

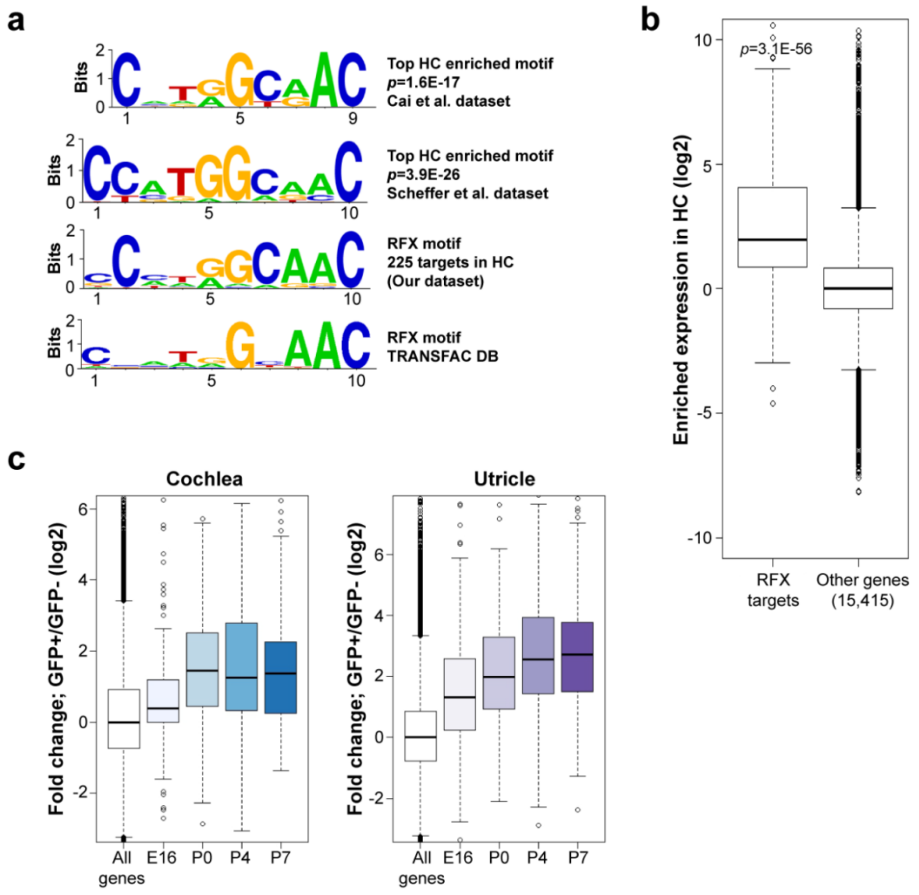

Supplementary Figure 8. RFX target genes are significantly enriched in two independent inner-ear HC RNA-seq datasets. Cai et al. and Scheffer et al. recently published two RNA-seq datasets that too, profiled the inner-ear HC transcriptome compared to non-HC populations[4,5]. **a.** In these two datasets too, the RFX signature was the most highly over-represented motif detected in the promoters of genes encoding HC-transcripts (hypergeometric test). For the dataset of Cai et al., a promoter analysis was applied to the set of genes whose expression was at least 2-fold elevated in cochlear HCs compared to Non-HCs; For the dataset of Scheffer et al., differential genes were first identified using ANOVA with GFP status (positive or negative) and organ (cochlea or utricle) as factors, followed by a promoter analysis to the set of genes which showed at least 2-fold increased expression in GFP(+) samples compared to GFP(-) ones. Shown are the DNA motifs detected in the promoters of HC-enriched genes from Cai et al., Scheffer et al.; in the promoters of the set of 225 putative RFX HC targets derived from our combined microarray-RNA-seq dataset, and the known RFX motif (TRANSFAC DB). **b.** The expression of the set of putative RFX target genes that derived from our combined microarray-RNA-seq dataset is significantly enriched in HCs from the dataset of Cai et al. (*P*-value calculated using Wilcoxon's test) **c.** The expression of the set of putative RFX target genes that derived from our combined microarray-RNA-seq dataset is enriched and continues to increase during development in both the auditory and vestibular HCs, as measured in the datasets of Scheffer et al. In this analysis, for each developmental time-point, expression ratios were calculated between the GFP(+) and GFP(-) samples. As a control, the “all genes” boxplot shows ratios calculated over all genes in the dataset for the P7 time-point; in all time-points the set of putative RFX target genes showed significantly elevated expression in GFP(+) cells; *p*-values range from 1.0E-10 to 1.E-58 using Wilcoxon test. For panels b and c, the boxes indicate the 1st and 3rd

quartiles; the horizontal band inside the box indicates the median. The whiskers extend to the most extreme data point which is no more than 1.5-times the interquartile range from the box.

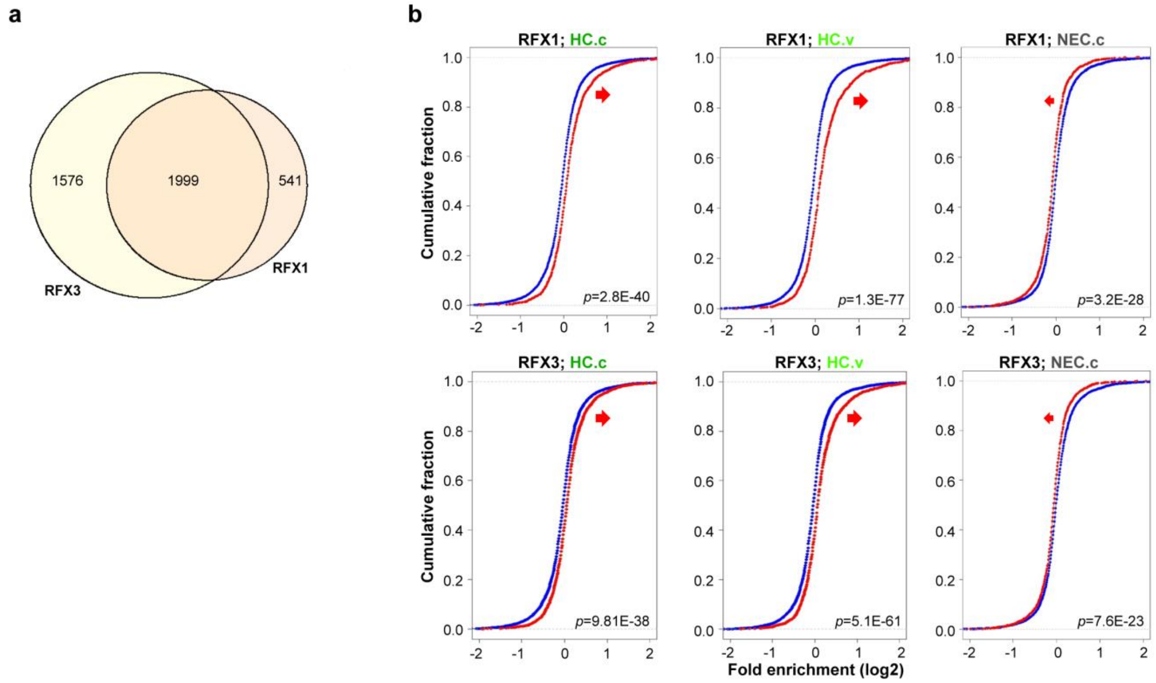

Supplementary Figure 9. ChIP-seq confirms that the expression of RFX target genes is significantly elevated in HCs. a. Venn-diagram showing significant overlap between target genes of RFX1 and RFX3, identified by ChIP-seq analysis carried out in the Min6 cell line. The high degree of overlap indicates a potential functional redundancy between RFX members. **b.** The analysis shown here is similar to the one shown in **Fig. 2d**. Ratios between expression level in the indicated cell-type and average expression in all three cell-types (HC, ENHC and NEC) were calculated for each gene in the microarray dataset. The plots compare the cumulative distribution of these ratios (in log2 scale; X-axis) between the set of RFX targets (red) and all the other genes in the dataset (blue). Deviation of the red curve to the right of the blue one indicates elevated expression of RFX targets, and vice versa. *p*-values were calculated using Wilcoxon test. Shown here are the results for RFX1 (top) and RFX3 (bottom). The red arrow indicates if the RFX1 targets, as a set, increased (to the right) or decreased (to the left) in their expression, compared with all other genes.

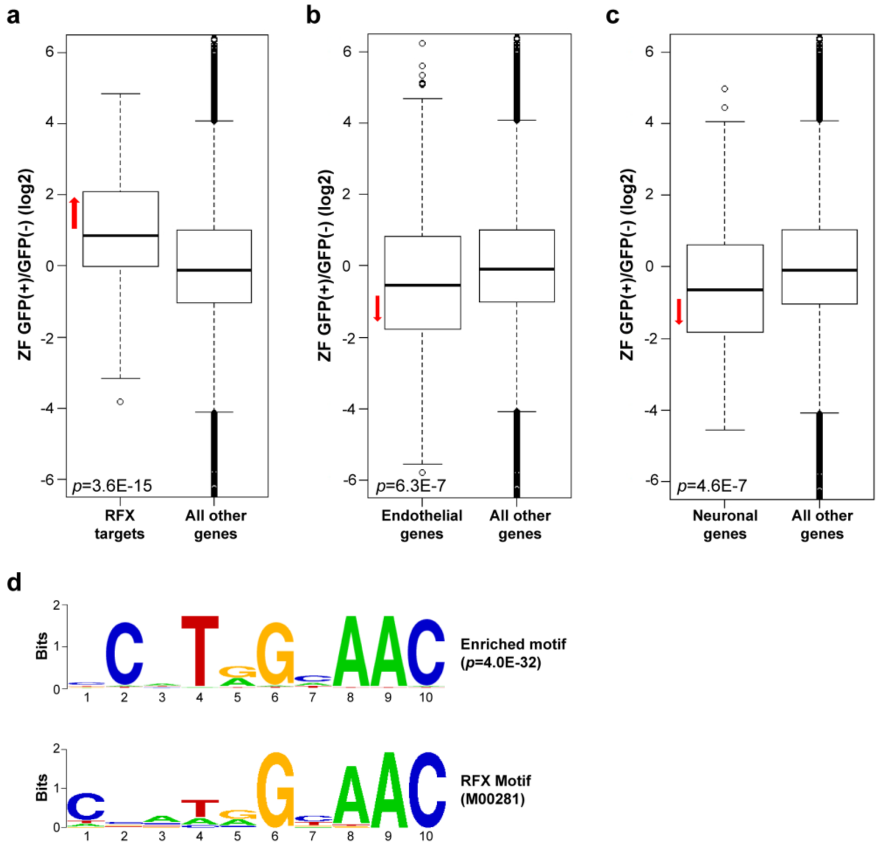

Supplementary Figure 10. The expression of zebrafish orthologous genes of the set of mouse putative RFX target genes is elevated in zebrafish HCs. a. Expression of the zebrafish orthologs of the 225 putative targets of RFX in murine HCs is significantly elevated in zebrafish HCs (GFP(+) cells). Ratios between expression levels in the GFP(+) and GFP(-) cells were calculated for each gene in the zebrafish RNA-seq dataset. The figure compares the distribution of these ratios (in log2 scale; Y-axis) between the set of orthologs of the RFX targets (left boxplot) and all the other genes in the dataset (right box-plot). **b and c.** Similar to (a) but testing, instead of RFX targets, zebrafish orthologs of murine genes whose expression was elevated in vascular endothelial and neuronal inner ear cells[2]. Unlike the expression of the RFX targets, the expression of the zebrafish orthologs of endothelial and neuronal genes is decreased in the GFP(+) cells, further demonstrating the specificity of our result. In panels a-c, *P*-values were calculated using Wilcoxon test. The boxes indicate the 1st and 3rd quartiles; the horizontal band inside the box indicates the median. The whiskers extend to the most extreme data point which is no more than 1.5-times the interquartile range from the box. **d.** *De-novo* motif analysis detected one significantly over-represented DNA motif (*p*-value=4.0E-32, hypergeometric test) in the promoters of the zebrafish genes encoding HC-enriched transcripts as defined by Steiner et al[3]. The results are based on the top 800 zebrafish genes with enriched transcripts in HCs. In this case too, the enriched motif matches the RFX binding signature.

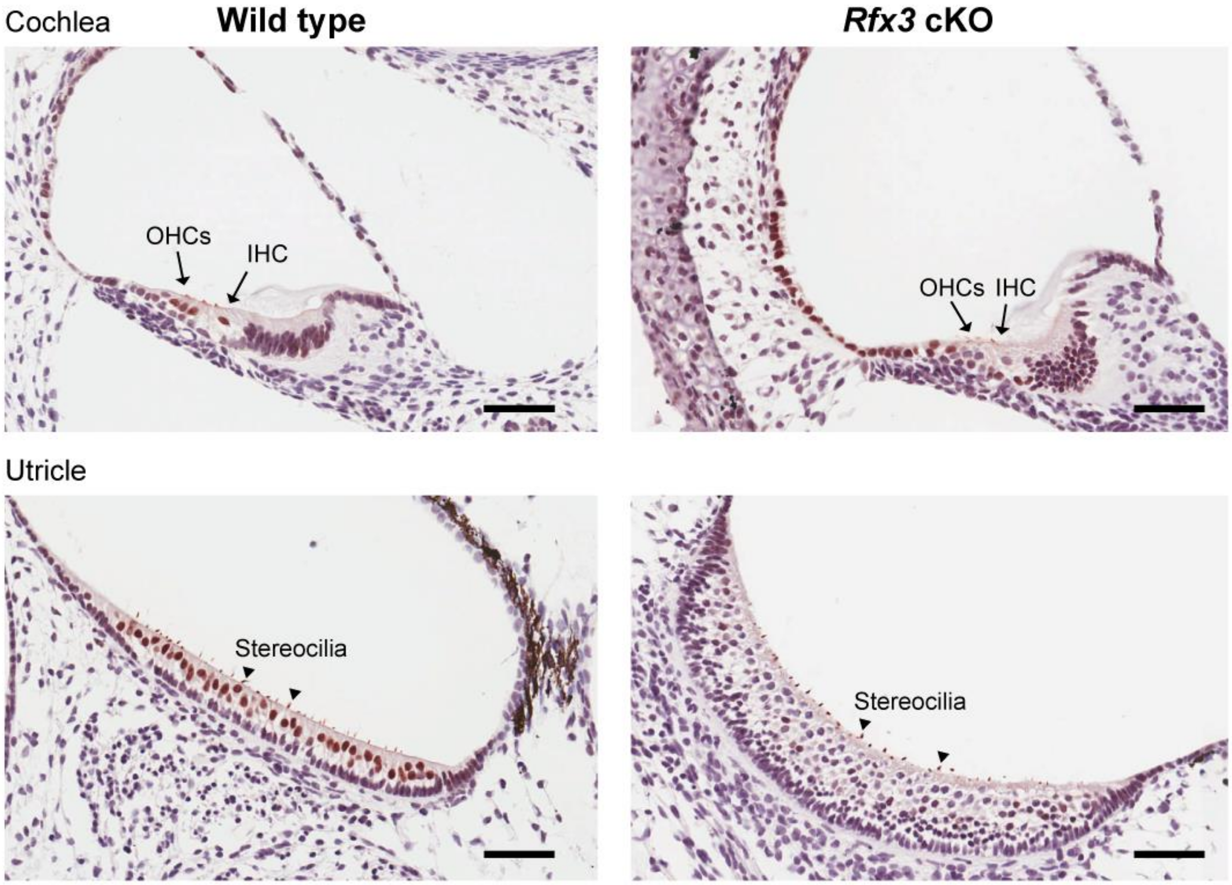

Supplementary Figure 11. Specific staining of HC nuclei by the RFX3 antibody. Inner ear sections from P1 wild type and *Rfx3* cKO mice, in which *Rfx3* was deleted from the inner ear HCs. Sections were stained with an antibody for RFX3 (brown) and then stained with H&E. Left panel – all HC nuclei stained positive for RFX3 in the wild type auditory and vestibular systems. Right panel – the expression of RFX3 is abolished from the HCs in the *Rfx3* cKO mice. The expression in the ENHCs is unchanged. The staining of the stereocilia is non-specific. Scale bar – 50 µm. The immune staining was performed as described in the Methods (Immunohistochemistry on paraffin sections) except that the RFX3 antibody was detected using the ImmPRESS™ REAGENT Anti-Rabbit Ig (Vector Laboratories) followed by incubation with the ImmPACT™ DAB Peroxidase Substrate (Vector Laboratories)

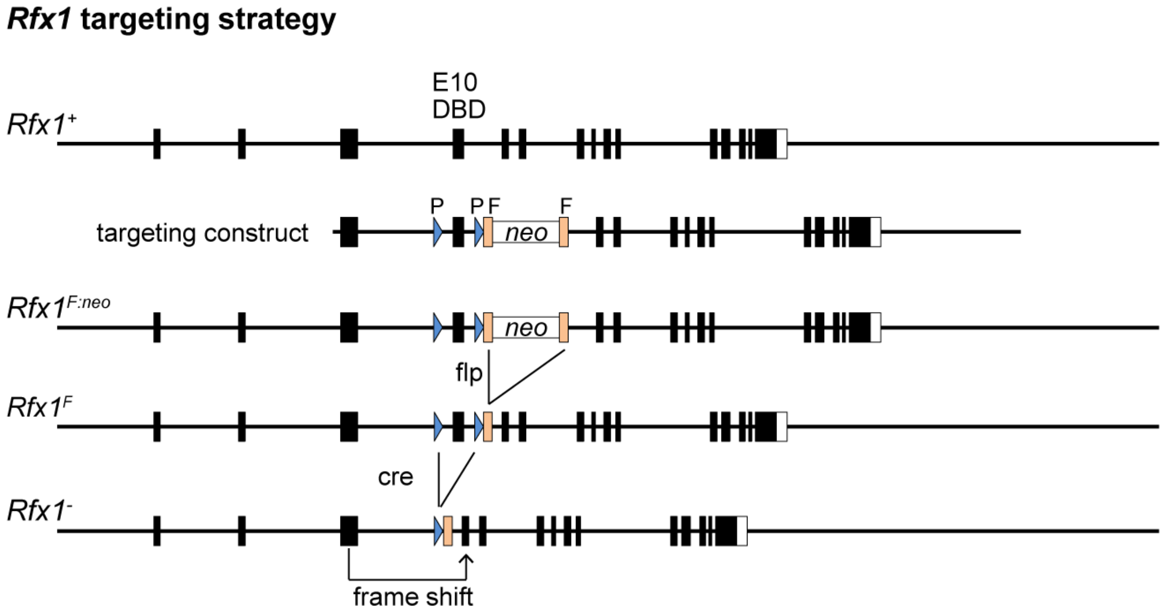

Supplementary Figure 12. *Rfx1* cKO strategy. A construct targeting exon 10 of the *Rfx1* DNA binding domain was generated by flanking exon 10 with two loxP sites and insertion of a frt (F) flanked neomycin resistance (pgk-neo-pA) cassette in the intron downstream of exon 10. Top line: genomic organization of the *Rfx1* gene. Second line: targeting construct. Third line: the *Rfx1* gene following recombination with the targeting construct. Fourth line: the *Rfx1 gene* after excision of the neomycin resistance cassette. Bottom line: the *Rfx1* gene following treatment with Cre-recombinase. *Rfx1*$^{+}$, WT locus. *Rfx1*$^{F:neo}$, loxP-flanked (floxed) locus retaining the neo cassette. *Rfx1*^{F}, loxP-flanked (floxed) locus. *Rfx1*$^{-}$, deleted locus.

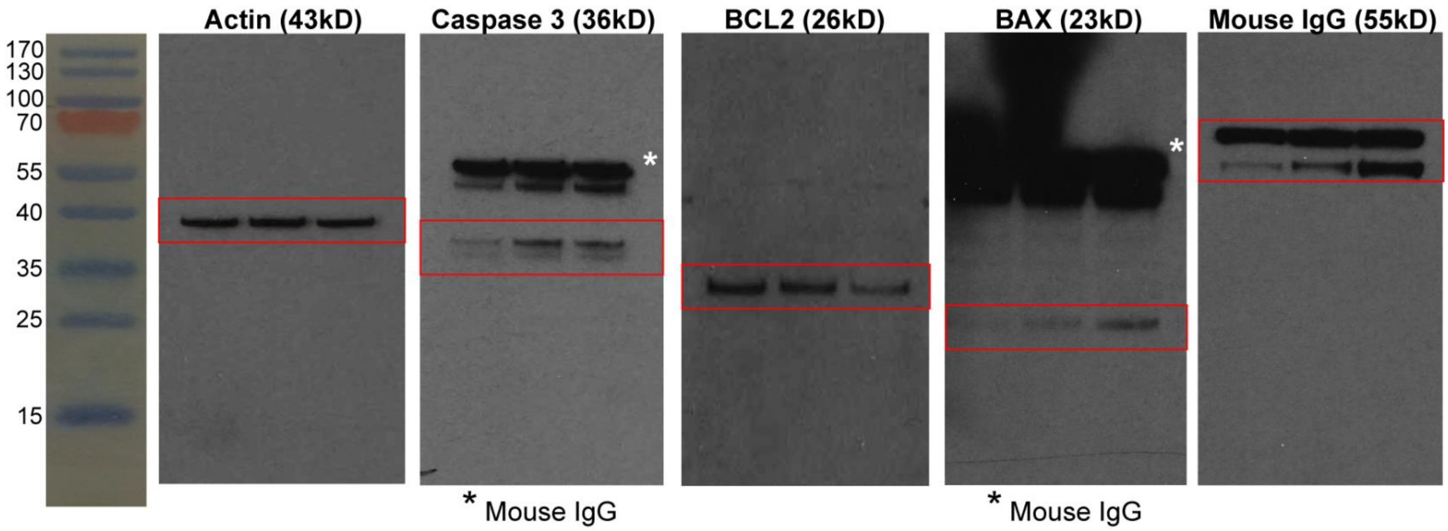

Supplementary Figure 13. Full blots used for Fig. 4. The red boxes represent the area of each blot used in Fig. 4. * in caspase 3 and BAX blot are mouse IgG heavy chain as shown in the last blot on the right blotted with only the secondary HRP antibody.

Supplementary References

1. Wasserman, W.W. & Sandelin, A. Applied bioinformatics for the identification of regulatory elements. *Nat Rev Genet* **5**, 276-87 (2004).
2. Hertzano, R. *et al.* Cell type-specific transcriptome analysis reveals a major role for Zeb1 and miR-200b in mouse inner ear morphogenesis. *PLoS Genet* **7**, e1002309 (2011).
3. Steiner, A.B., Kim, T., Cabot, V. & Hudspeth, A.J. Dynamic gene expression by putative hair-cell progenitors during regeneration in the zebrafish lateral line. *Proc Natl Acad Sci U S A* **111**, E1393-401 (2014).
4. Cai, T. *et al.* Characterization of the transcriptome of nascent hair cells and identification of direct targets of the Atoh1 transcription factor. *J Neurosci* **35**, 5870-83 (2015).
5. Scheffer, D.I., Shen, J., Corey, D.P. & Chen, Z.Y. Gene Expression by Mouse Inner Ear Hair Cells during Development. *J Neurosci* **35**, 6366-80 (2015).
